# Crosstalk between m6A and coding/non-coding RNA in cancer and detection methods of m6A modification residues

**DOI:** 10.18632/aging.204836

**Published:** 2023-07-11

**Authors:** Qingren Meng, Heide Schatten, Qian Zhou, Jun Chen

**Affiliations:** 1 National Clinical Research Center for Infectious Diseases, Shenzhen Third People’s Hospital, The Second Hospital Affiliated with the Southern University of Science and Technology, Shenzhen, Guangdong Province, China; 2 Department of Veterinary Pathobiology, University of Missouri, Columbia, MO 65211, USA; 3 International Cancer Center, Shenzhen University Medical School, Shenzhen, Guangdong Province, China

**Keywords:** N6-methyladenosine (m6A), RNA modification, detection method, m6A database, tumor

## Abstract

N6-methyladenosine (m6A) is one of the most common and well-known internal RNA modifications that occur on mRNAs or ncRNAs. It affects various aspects of RNA metabolism, including splicing, stability, translocation, and translation. An abundance of evidence demonstrates that m6A plays a crucial role in various pathological and biological processes, especially in tumorigenesis and tumor progression. In this article, we introduce the potential functions of m6A regulators, including “writers” that install m6A marks, “erasers” that demethylate m6A, and “readers” that determine the fate of m6A-modified targets. We have conducted a review on the molecular functions of m6A, focusing on both coding and noncoding RNAs. Additionally, we have compiled an overview of the effects noncoding RNAs have on m6A regulators and explored the dual roles of m6A in the development and advancement of cancer. Our review also includes a detailed summary of the most advanced databases for m6A, state-of-the-art experimental and sequencing detection methods, and machine learning-based computational predictors for identifying m6A sites.

## INTRODUCTION

**Figure 1:**
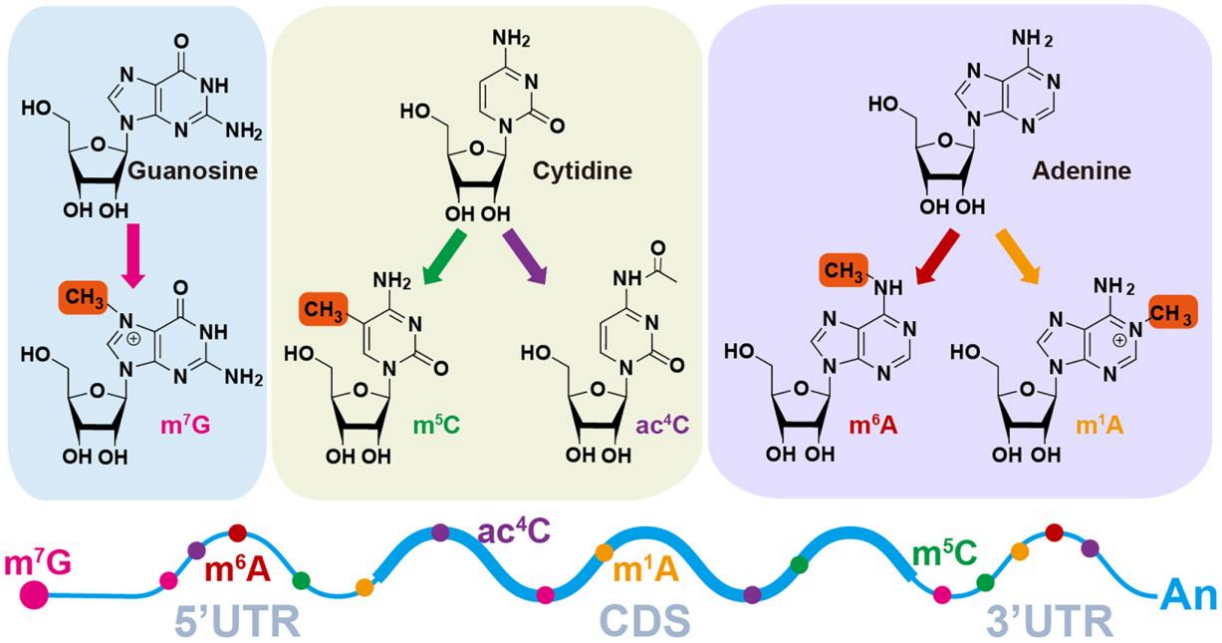
**Diverse RNA modification patterns.** m7G, N7-methylguanosine; m6A, N6-methyladenosine; ac4C, N4-acetylcytidine; m1A, N1-methyladenosine; m5C, N5-methylcytidine.

As is well known, RNAs play an essential role in the translation process, gene regulation, and environmental interactions [1]. Chemical modification is a highly specific and effective method for regulating the functions of biological macromolecules. These macromolecules can be covalently modified after synthesis, such as RNA, DNA, protein, lipids, carbohydrates, and polysaccharides [2]. RNA modification not only has dynamic reversibility but can also be regulated by a wide range of factors, which has led to the emergence of the term “RNA epitranscriptome” and has become increasingly popular in recent years [3]. Recently, more than 100 RNA modification types on bases or ribose have been discovered within all types of RNA, including ribosome RNA (rRNA), transfer RNA (tRNA), messenger RNA (mRNA) and non-coding RNA (ncRNA), which have been confirmed to be involved in many diseases and bio-processes [2, 4, 5]. For example, various RNA modifications in mRNA like N6-methyladenosine (m6A) [6], N4-acetylcysteine (ac4C) [7], N7-methyl guanidine (m7G) [8], 5-methylcytidine (m5C) [9] and N1-methyladenosine (m1A) [10] (Figure 1), are conducive to RNA metabolism steps including splicing, translocation, transcript stability and translation in eukaryotic cells, especially m6A [11]. NAT10 (RNA acetyltransferase)-mediated ac4C can promote mRNA’s stability by extending the mRNA’s half-life in Hela cells [12]. In addition, METTL1-dependent m7G on pre-let-7 regulates pre-let-7 and let-7 expression levels and lung cancer cell migration [13]. Interestingly, even when targeting the same type of methylation, the sets of methyltransferases and demethylases, acting as writers and erasers respectively, can vary across different types of RNA species [14].

This review will summarize the physiological function of m6A or m6A regulators and focus on the interplay of m6A and protein-coding RNA, and ncRNAs (miRNA, circRNA, lncRNA) in various cancers. Finally, we will list the experimental detection and prediction methods of m6A modification residues and summarize m6A-related databases.

## Functional mechanism of m6A modification

**Figure 2:**
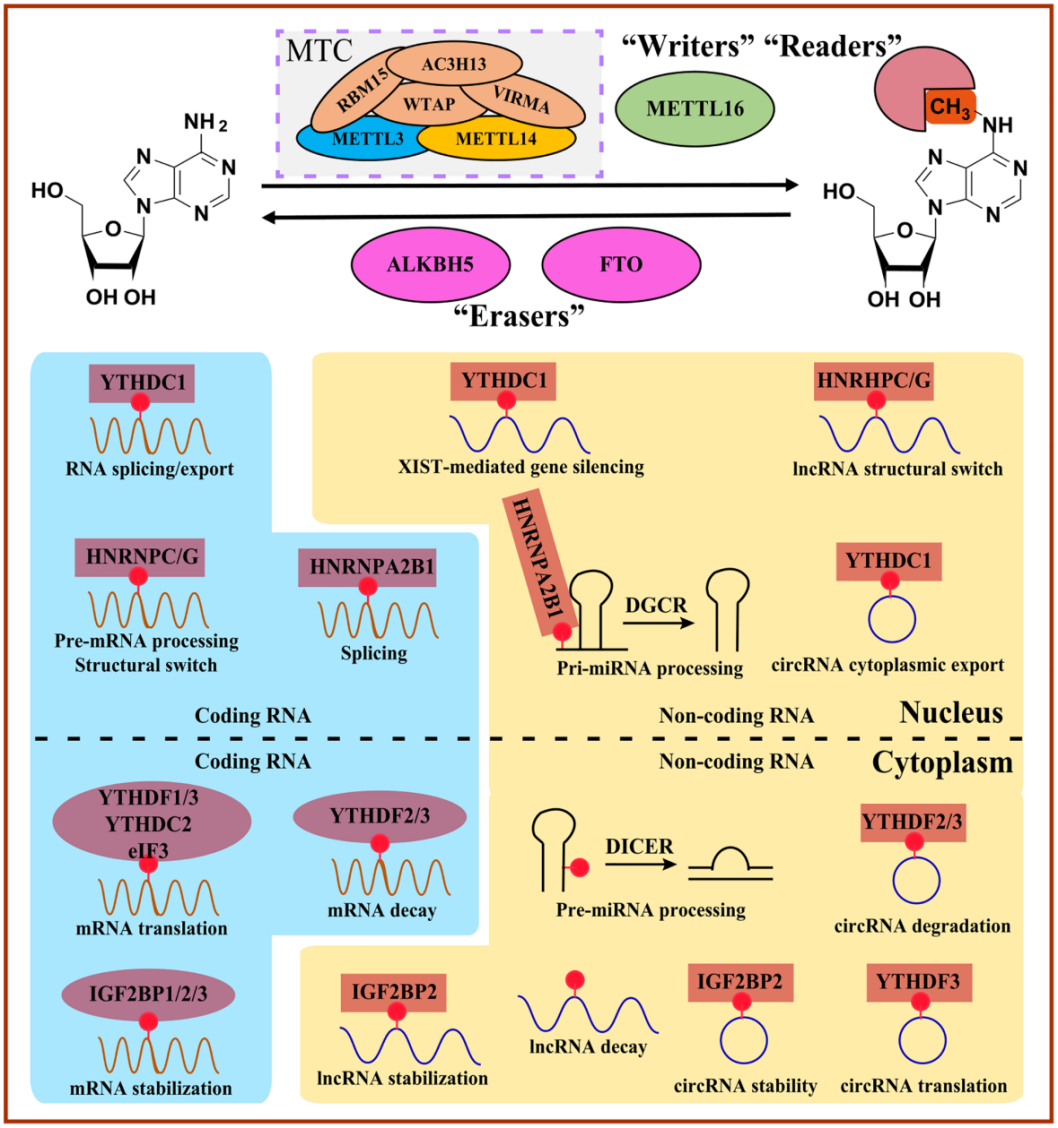
**Overview of m6A-mediated RNA metabolism.** “Writers” install m6A on coding RNAs and non-coding RNAs, whereas m6A can be reversibly removed by “Erasers”. Diverse m6A “Readers” determine the fate of m6A-modified RNAs involved in the structural switch, splicing, translocation, translation, and decay. MTC, methyltransferase complex.

The enzymes that deposit, remove and bind to RNA modifications are writers, erasers, and readers [3]. Intriguingly, m6A modifications are reversible and dynamic, which is mediated by catalyzing writers and erasers (Table 1). Generally, adenosine methylation occurs preferentially within different consensus sequences. The METTL3/14 complex prefers RRACH (R = G or A; H = A, C or U) in coding regions and 3′UTRs or consensus motif DRACH (D = A, G or U). The crystal structure suggests that another writer, METTL16, prefers a UAC (m6A) GAGAA motif in the bulge of a stem-loop structured RNA (Figure 2, Table 1) [11, 15–17].

**Table 1:** Biological functions of m6A regulators in RNA metabolism.

**Type**	**Regulators**	**Location**	**Functions**	**References**
m6A writers	METTL3	Nucleus	Installs m6A on mRNAs as well as lncRNAs and mediate RNA translation	[11, 183]
	METTL14	Nucleus	Assists METTL3 for m6A deposition and strengthens METTL3 activity	[11, 15, 184]
	METTL16	Nucleus	Installs m6A in U6 snRNA	[26]
	WTAP	Nucleus	Mediates METTL3-METTL14 to the nuclear speckle	[21]
	VIRMA	Nucleus	Guides the writers to RNA specific region	[23]
	RBM15	Nucleus	Binds to m6A complex and recruit it to special RNA site	[22]
	ZC3H13	Nucleus	Assists WTAP location to Nito	[185]
m6A eraser	FTO	Nucleus	Removes m6A modification and mediates alternative splicing	[27]
	ALKBH5	Nucleus	Removes m6A modification	[29]
m6A readers	YTHDF1	Cytoplasm	Promotes RNA translation	[20]
	YTHDF2	Cytoplasm	Reduces RNA stability and facilitates RNA degradation	[36]
	YTHDF3	Cytoplasm	Mediates the translation with YTHDF1 and strengthens RNA decay with YTHDF2	[39]
	YTHDC1	Nucleus	Promotes RNA alternative splicing and regulate nuclear mRNA transport	[35]
	YTHDC2	Cytoplasm	Augments RNA translation	[186]
	HNRNPA2B1	Nucleus	Promotes primary microRNA processing and regulates alternative splicing	[42]
	HNRNPC	Nucleus	Participates in pre-mRNA splicing	[43, 187, 188]
	HNRNPG	Nucleus	Mediates pre-mRNA splicing	[187]
	IGF2BP1	Cytoplasm	Enhances mRNA stability and translation	[47]
	IGF2BP2	Cytoplasm	Enhances mRNA stability and translation	[47]
	IGF2BP3	Cytoplasm	Enhances mRNA stability and translation	[47]

### Writers

The m6A modifications can be catalyzed by m6A writers (m6A methyltransferases). Typically discovered writers include methyltransferase-like 3/14/16 (METTL3/14/16) and other cofactors such as RNA binding motif protein 15/15B (RBM15/15B), wt1-associated protein (WTAP) and vir-like m6A methyltransferase-associated protein (VIRMA), which form the methyltransferase complex (MTC) [11]. Among them, METTL3 is a core component with methyltransferase activity by transferring the methyl from S-adenosyl methionine (SAM) into the N6 site of adenosine [11]. In comparison, METTL14 can impact the stability and methyltransferase activity [18] of the METTL3 complex and recognize the histone modification mark H3K36me3 to regulate the selection of modification sites on RNA [19, 20]. WTAP, known as cofactors and regulatory components of the complex, can guide METTL3/14-RNA binding and locate into nuclear speckles [21]. And RBM15 and its paralog RBM15B show the WTAP-dependent function of recruiting MTC into specific methylate RNA sites [22]. VIRMA is considered an essential part of MTC, showing a similar function as RBM15 [23]. Loss of *METTL3* will induce endothelial cells to fail to transform into hematopoietic stem/progenitor cells in zebrafish and mouse embryos [24]. In addition, specific deletion of *METTL3* in the central nervous system can lead to severe motif motor dysfunction during lactation and death in mice [25].

Other atypical methyltransferases such as METTL16, ZCCHC4, and METTL5 are discovered and indispensable for m6A [5]. For example, METTL16, a conserved U6 snRNA methyltransferase, regulates the intracellular SAM contents by methylating the SAM synthetase gene MAT2A and participating in the RNA splicing process [26]. Su and colleagues found that METTL16 had the most crucial effect on the survival of tumor cells among all METTL protein families. *METTL*16 plays a more critical role in almost all tumor cells as an oncogenic gene compared to *METTL3* and *METTL14*. In the nucleus, METTL16 catalyzed the m6A modification of mRNA, especially of nascent mRNA. In the cytoplasm, METTL16 promoted the assembly of 80S ribosomes by directly binding with eIF3a/b and rRNAs, thus improving the translation efficiency of proteins and thus promoting the development of tumors (such as liver cancer) [18]. These results suggest that targeting METTL16, primarily the Mtase domain, may be a potential new tumor therapy strategy.

### Erasers

m6A erasers account for the reverse demethylation of modified m6A base, which belongs to the Fe(II)/α-KG-dependent dioxygenase AlkB family: α-ketoglutarate-dependent dioxygenase AlkB homolog 5 (ALKBH5) and the mass-and obesity-associated protein (FTO) [5, 11]. Although FTO is the first identified demethylating enzyme associated with adipogenesis, the following evidence indicates that FTO preferentially demethylates 2’-O-dimethyladenosine (m6Am) [27]. More recently, FTO was revealed to demethylate N1-methyladenosine (m1A) in tRNA [28]. ALKBH5, considered as a secondly discovered m6A demethylase enzyme, has the conservatively catalytic domain to recognize and demethylate m6A modification sites regardless of single or double-stranded RNA [5]. *ALKBH5* knockdown in mice affects nascent mRNA synthesis and the splicing rate in mice and humans, respectively [29, 30].

### Readers

m6A modification can be recognized by the translation initiation factor eukaryotic initiation factor 3 (eIF3), heterogeneous nuclear ribonucleoproteins (HNRNPs), insulin-like growth factor 2 mRNA-binding proteins (IGF2BPs), and proteins containing the YT521B homology (YTH) domain. In addition, specific m6A readers may mediate different physiological processes, such as immune response and biological rhythm, and participate in RNA metabolism steps such as RNA degradation and translation by binding the m6A modification site directly [11, 31, 32].

Generally, the YTH domain family is composed of YTH domain family protein 1-3 (YTHDF1-3, DF family) and YTH domain-containing protein 1-2 (YTHDC1-2, DC family) in mammalian cells. YTHDC1 belongs to nuclear m6A readers functioning on selectively binding m6A-containing precursor RNAs, while other YTH family members, including YTHDF1, YTHDF2, YTHDF3, and YTHDC2, identified as cytoplasmic m6A readers, regulate the mature mRNAs containing m6A [33].

YTHDC1 also can recognize m6A on XIST and enhance XIST-mediated X chromosome silencing [22]. YTHDC1, in Hela cells, recognizes m6A to form mRNA-protein complex and further interacts with RNA export factor 1 (NXF1) and serine/arginine-rich splicing factor 3 (SRSF3) to facilitate the m6A-methylated mRNAs nuclear export [34]. In addition to the function of silencing and nuclear export, YTHDC1 promotes exon inclusion or exon skipping via selectively recruiting or inhibiting different splicing factors, such as serine-arginine repeat protein. In addition, YTHDC1 recruits competitive RNA splicing factors SRSF3 and blocks serine/arginine-rich splicing factor 10 (SRSF10) from binding to mRNA to regulate exon inclusion and exon skipping, respectively [35].

YTHDC2 preferentially binds m6A within a precisely consensus motif and promotes the translation efficiency of mRNAs. YTHDC2 contains multiple functional elements, such as ankyrin repeats and the R3H domain, and these unique features endow YTHDC2 with many functions, including regulatory effects on RNA binding and structure and recruitment of or binding with other protein complex members [33]. YTHDF1, in Hela cells, can bind to mRNA stop codons and m6A near the 3′UTR region, and augment translation efficiency by interacting with eukaryotic initiation Factor 3 (eIF3) in the 48S translation initiation complex rather than by the m7G-cap-dependent manner [20].

YTHDF2 acts as an m6A reader inducing mRNA degradation [36]. YTHDF2 decodes m6A on more than 2000 mRNAs in HeLa cells. And YTHDF2 knockout can cause the abundance of these mRNAs to increase significantly, suggesting that YTHDF2 is essential for m6A-modified mRNAs [36]. Furthermore, the stability of mRNAs regulated by YTHDF2 positively correlates with the degree of polyadenylation at its 3’end. mRNAs combined with YTHDF2 generally exhibit shorter polyadenylic acid tails than other mRNAs. Typically, YTHDF2 first binds to m6A through the YTH domain, and then its N-terminal domain interacts with the SH (superfamily homolog) domain of CNOT1 and recruits the CCR4/CAF/NOT complex to perform deadenylation [37]. Intriguingly, YTHDF2 is thought to co-localize with both decapping and deadenylation enzyme complexes under normal conditions and directs its target RNAs to processing bodies [37]. However, recent studies showed that almost no significant mRNA degradation events were observed in the p-body, indicating the importance and significance of YTHDF2 in directing the process of RNAs into p-body that still needs to be considered and explored [38]. YTHDF2 can also enhance the expression level of pro-inflammatory cytokines by promoting the degradation of KDM6B, known as the repressive mark H3K27me3 demethylase. Meanwhile, the repressive mark H3K27me3 can inhibit the methylation process on adenosine during the transcription process. KDM6B recruits m6A methyltransferase to remove the H3K27me3 modification near the target gene and promotes the m6A modification on the newly transcribed mRNA, suggesting that complex interactions of m6A modification and histone modification exist and need to be studied [19].

YTHDF3, together with YTHDF1, guides the m6A modified transcript to be combined with YTHDF2 to direct the target RNA into RNA degradation steps. In addition, YTHDF3 could mediate mRNA decay by interacting with YTHDF2. YTHDF3 could cooperate with YTHDF3 to regulate mRNA translation; however, it could not function directly [39]. Moreover, YTHDF3 deficiency will reduce the binding ability of YTHDF1 and YTHDF2 to the target RNA, while YTHDF1 or YTHDF2 loss reduces the amount of RNA that YTHDF3 binds, suggesting that YTHDF3 could indirectly improve m6A-dependent translation efficiency by enhancing the binding ability for YTHDF1 and its target RNAs. Intriguingly, the translation process of circRNA with m6A modification does not depend on YTHDF1 at all, but YTHDF3 is required. Notably, YTHDF3 recruits eIF4G2 that directly binds to internal ribosome entry sites (IRES) to initiate the translation process independent of eIF4E [40, 41].

HNRNP family members, a well-known RNA-binding protein family, involved in m6A modification, consist of heterogeneous nuclear ribonucleoproteins A2/B1 (HNRNPA2B1), heterogeneous nuclear ribonucleoproteins G (HNRNPG) and heterogeneous nuclear ribonucleoproteins C (HNRNPC). Controversially, is HNRNPA2B1 an m6A reader or not? One study showed that HNRNPA2B1 could directly bind m6A and regulate alternative splicing events by cooperating with METTL3 and primary microRNA processing via interacting with pri-miRNA microprocessor complex component DGCR8. In contrast, a structure-based study revealed an “m6A switch” mechanism rather than specific m6A binding mediated by HNRNPA2B1 [42]. Another two HNRNP family members, HNRNPC and HNRNPG, can regulate the processing of m6A-containing RNA transcripts. HNRNPC also participates in the pre-mRNAs processing steps [43]. However, they do not bind to m6A directly, suggesting that HNRNPC and HNRNPG can bind to the transcripts via RNA structure alternations determined by m6A [44].

eIF3, considered as an m6A reader, recruits 43S protein complex to initiate the translation steps for mRNAs containing 5′UTR m6A without cap-binding factor eIF4E, that is, eIF3 functions in a cap-dependent manner. However, the exact mechanism of eIF3 for m6A recognition is not yet clearly understood. It may rely on the sequence motif like YTH proteins and adjacent RNA structure [45, 46].

IGF2BPs are the conserved m6A-binding proteins, whose RNA-binding domains are composed of RNA recognition motif (RRM) domains and K homology (KH) domains. IGF2BP family members, IGF2BP1, IGF2BP2, and IGF2BP3, can promote the stability and increase the translation efficiency of m6A-modified mRNAs. Additionally, the mRNA stabilization regulated by IGF2BPs may be enhanced by recruiting cofactors such as matrin 3 (MATR3) and ELAV-like RNA binding protein 1 (ELAVL1) [47].

## m6A writers, erasers, and readers in cancers

**Figure 3:**
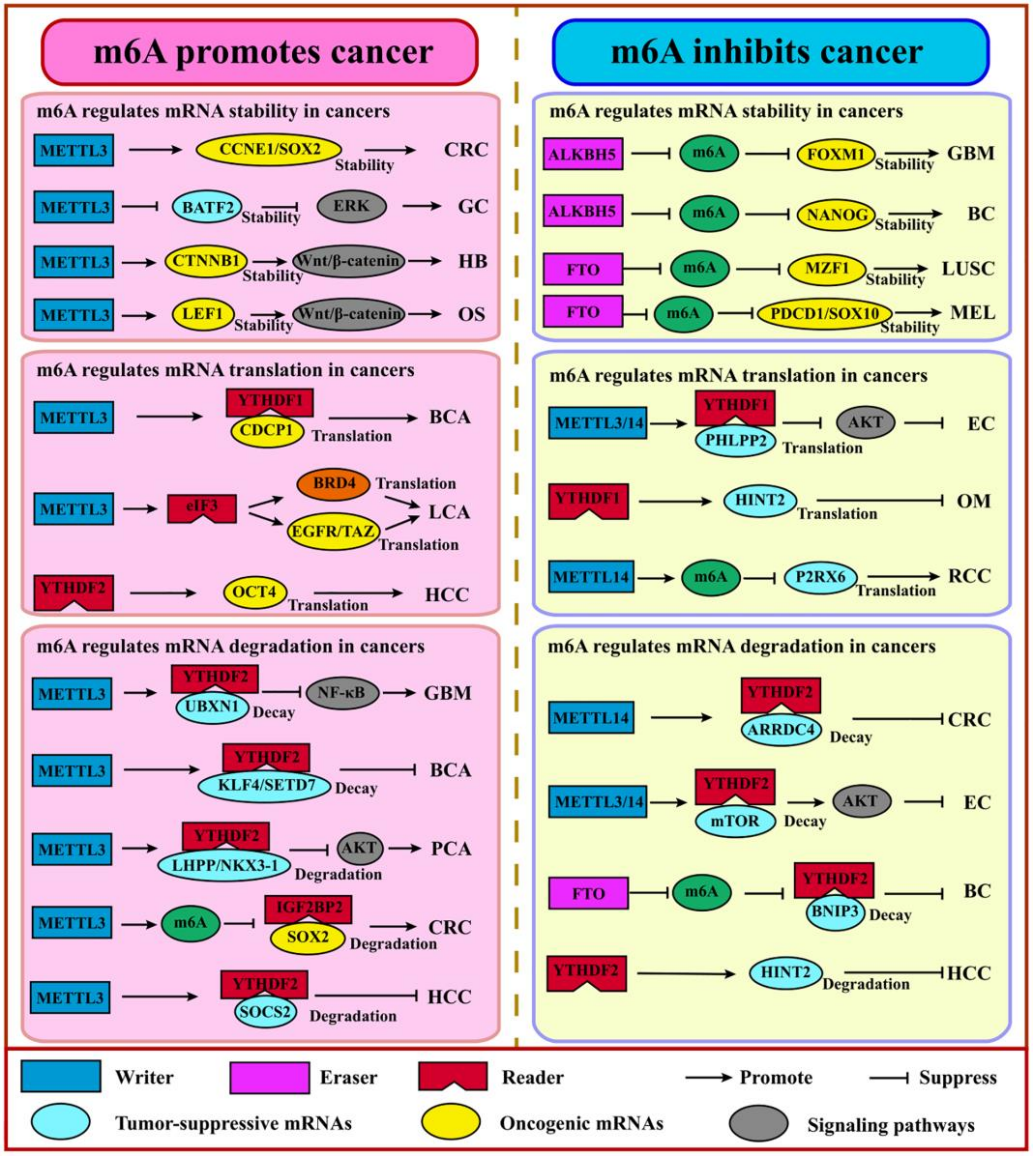
**m6A regulates coding RNAs in cancers.** Generally, m6A promotes cancer via the stabilization or translation of oncogenes and the degradation of tumor suppressor genes. Adversely, m6A suppresses cancer via the stabilization or translation of tumor suppressor genes, the degradation of the oncogene. Abbreviations: GBM: glioblastoma; GC: gastric cancer; HB: hepatoblastoma; OS: osteosarcoma; BCA: bladder cancer; LCA: lung cancer; HCC: hepatocellular cancer; PCA: prostate cancer; CRC: colorectal cancer; BC: breast cancer; LUSC: lung squamous cell carcinoma; MEL: melanoma; OM: ocular melanoma; RCC: renal cell carcinoma; EC: endometrial carcinoma.

Growing studies revealed that the global abundance of m6A and expression level of m6A regulators often show aberrance across various cancers and are associated with cancer initiation, progression, metastasis, relapse, and therapy [48–50]. Human HCC showed a higher abundance of m6A and augmented gene expression levels when YTHDC2 was knocked down, whereas the global abundance of m6A was reduced significantly in human bladder cancer [51]. This contradiction suggests that research on m6A needs to be more detailed and context dependent. Thus, a comprehensive understanding of m6A regulators may account for better prevention and treatment (Figure 3, Table 2).

**Table 2:** Aberrant m6A regulators and its potential mRNA targets in cancers.

**m6A regulator**	**Cancer type**	**Regulators Roles**	**Target**	**Regulators functions**	**References**
METTL3-METTL14	Acute Myelocytic Leukemia	Oncogene	MYC, SP1, SP2, BCL2, PTEN	↑Proliferation	[189]
	Lung cancer	Oncogene	EGFR, BRD4, JUNB	↑Invasion, ↑Proliferation	[59, 60, 190]
	Hepatocellular carcinoma	Oncogene	SOCS2, SNAIL	↑Proliferation, ↑Migration	[54, 191]
	Endometrial cancer	Tumor suppressor gene	PHLPP2	↓Proliferation, ↓Migration	[192]
	Pancreatic cancer	Tumor suppressor gene	PHLPP2	↓Proliferation	[64]
	Pancreatic cancer	Oncogene	PERP	↑Growth, ↑Metastasis	[193]
	Glioblastoma	Oncogene	SOX2, SRSF, ADAR1	↑self-renewal, ↑Proliferation	[52, 194, 195]
	Glioblastoma	Tumor suppressor gene	ADAM19, KLF4, BRCA2, CDKN2A	↓Growth, ↓self-renewal	[63]
	Ovarian tumor	Oncogene	AXL	↑Invasion	[196]
	Bladder cancer	Oncogene	AFF4, IKBKB, CDCP1, MYC, ALF4	↑Growth	[57, 58, 197]
	Breast cancer	Oncogene	MYC, KLF4, HBXIP	↑Proliferation, ↑Migration	[198, 199]
	Breast cancer	Tumor suppressor gene	NOTCH1	↓Proliferation	[152]
	Prostate cancer	Oncogene	LEF1/LHPP	↑Proliferation, ↑Migration	[200, 201]
	Colorectal cancer	Tumor suppressor gene	ARRDC4	↓Invasion, ↓Migration	[202]
	Colorectal cancer	Oncogene	CCNE1	↑Proliferation	[56]
	Thyroid carcinoma	Oncogene	TCF1	↑Proliferation	[203]
	Gastric cancer	Oncogene	BATF2, HDGF	↑Proliferation	[53, 204]
	Kidney cancer	Tumor suppressor gene	P2RX6	↓Invasion, ↓Migration	[205]
	Hepatoblastoma	Oncogene	CTNNB1	↑Proliferation	[55]
	Nasopharynegal carcinoma	Oncogene	ZNF750	↑Growth	[206]
WTAP	Hepatocellular carcinoma	Oncogene	ETS1	↑Proliferation	[67]
	Osteosarcoma	Oncogene	HMBOX1	↑Tumorigenicity	[207]
RBM15	Laryngeal squamous cell carcinoma	Oncogene	TMBIM6	↑Proliferation	[208]
FTO	Cutaneous melanoma	Oncogene	PDCD, CXCR4, SOX10	↑Tumorigenicity	[74]
	Acute Myelocytic Leukemia	Oncogene	ASB2, RARA, MYC, CEBPA	↑Proliferation	[71]
	Clear cell renal cell carcinoma	Tumor suppressor gene	PGC-1α	↓Proliferation	[209]
	Cervical cancer	Oncogene	β-catenin	↑Development	[210]
	Lung cancer	Oncogene	MZF1	↑Proliferation, ↑Invasion	[211]
	Hepatocellular carcinoma	Oncogene	PKM2	↑Tumorigenicity	[212]
	Breast cancer	Oncogene	BNIP3	↑Growth	[73]
ALKBH5	Glioblastoma	Oncogene	FOXM1	↑Tumorigenicity	[80]
	Acute Myelocytic Leukemia	Oncogene	TACC3, AXL	↑Self-renewal	[81, 213]
	Ovarian tumor	Oncogene	NANOG	↑Proliferation	[214]
	Pancreatic cancer	Tumor suppressor gene	PER1	↓Proliferation	[215]
	Lung cancer	Oncogene	UBE2C, TIMP3	↑Proliferation	[216, 217]
	Hepatocellular carcinoma	Tumor suppressor gene	LYPD1	↓Proliferation	[83]
YTHDC2	Lung adenocarcinoma	Tumor suppressor gene	SLC7A11	↓Tumorigenicity	[218]
	Bladder cancer	Oncogene	CDCP1	↑Growth	[197]
YTHDF1	Colorectal cancer	Oncogene	Wnt/β-catenin	↑Tumorigenicity	[87]
	Hepatocellular carcinoma	Oncogene	FZD5	↑Proliferation	[84]
	Non-small-cell lung cancer	Oncogene	CDK2, CKD4	↑Proliferation	[86]
	Gastric cancer	Oncogene	FZD7	↑Tumorigenicity	[219]
	Endometrial cancer	Tumor suppressor gene	PHLPP2	↓Proliferation	[192]
	Ovarian tumor	Oncogene	EIF3C	↑Proliferation, ↑Migration	[220]
	Ocular melanoma	Tumor suppressor gene	HINT2	↓Proliferation	[91]
YTHDF2	Glioblastoma	Oncogene	MYC/UBXN1	↑Proliferation, ↑Self-renewal	[221, 222]
	Ovarian cancer	Oncogene	BMF	↓Apoptosis	[223]
	Hepatocellular carcinoma	Oncogene	OCT4	↑Growth	[224]
	Hepatocellular carcinoma	Tumor suppressor gene	EGFR	↓Proliferation, ↓Growth	[94]
	Ovarian tumor	Oncogene	FOXK1, PDLIM7	↑Growth	[225]
	Liver cancer/ovarian cancer/lung cancer	Oncogene	E2F1	↑Proliferation	[226]
IGF2BP1	Colorectal cancer	Oncogene	SOX2, SEC62	↑Tumorigenicity, ↑Proliferation	[227, 228]
	Hepatocellular carcinoma	Oncogene	SRF/FEN1	↑Growth	[225, 226]
IGF2BP2	Clear cell renal cell carcinoma	Oncogene	CDK4	↑Proliferation	[229]
	Colon cancer	Oncogene	CCND1	↑Proliferation	[230]
IGF2BP3	Melanoma	Oncogene	MMP2	↑Proliferation, ↑Migration	[231]
	Multiple myeloma	Oncogene	ILF3	↑Growth	[232]

## Dysregulation of m6A writers in cancer

### METTL3

METTL3 mainly regulates the expression of oncogenes and tumor suppressor genes at the post-transcription level, including mRNA stability and the translation process. METTL3 induces growth enhancement and self-renewal of glioma stem cells. METTL3 is up-regulated in human glioblastoma tissues and induces m6A modification by binding to SOX2 mRNA in 3′UTR. The growth of murine glioblastoma can be decelerated or stagnated by silencing the METTL3 to inhibit the expression level of SOX2 [52]. Additionally, the overexpression of METTL3 and its oncogenic role has been revealed in various cancers such as gastric cancer [53], hepatocellular carcinoma [54], hepatoblastoma [55], colorectal cancer [56], bladder cancer [57, 58] and lung cancer [59, 60]. For example, METTL3 promotes HCC growth and accounts for HCC progression via repressing SOCS2 level. Moreover, higher expression of METTL3 is detected and explored to be associated with CRC metastasis and a poor prognosis [54]. And the higher METTL3 expression in cutaneous squamous cell carcinoma facilitates Np63 expression, which leads to rapid proliferation and growth of CSCC cells [61]. In pancreatic cancer cells, *METTL3* knockdown does not affect cell proliferation; however, it increases the sensitivity of cells to anticancer drugs such as gemcitabine and cisplatin [62].

### METTL14

Interestingly, METTL14 also plays dual roles in various cancers [52, 63]. The reduction of METTL14 promotes a vicious phenotype by up-regulating oncogene and down-regulating suppressor genes in glioblastoma [63]. Similar results are seen in endometrial cancer: the depletion of METTL14 induces higher cell proliferation by activating AKT pathways [64]. Adversely, METTL14 plays a pro-tumorigenic function in breast cancer by determining the m6A levels of EMT and angiogenesis-related transcripts such as *TWIST* [65]. In addition, in hematopoietic stem cells and AML, METTL14 deficiency promotes myeloid differentiation and inhibits self-renewal via decreasing m6A signals for targets *MYB* and *MYC* to reduce their stability and translation efficiency [66].

### WTAP

WTAP is overexpressed in HCC, which enhances tumor cell migration and invasion [67]. Notably, WTAP acts as an oncogene in AML [68]. Another component of the protein complex, VIRMA, can promote cancer cell proliferation and metastasis of breast cancer by regulating *CDK1* expression in an m6A-dependent manner [69]. Moreover, in liver cancers, overexpression of VIRMA facilitated cell invasion, and down-regulation of VIRMA inhibited the m6A modification for *ID2* or *GATA3* mRNAs [70].

## Dysregulation of m6A erasers in cancer

### FTO

FTO, acting as the m6A demethylase as first discovered, plays an oncogenic role in AML by promoting leukemogenesis via reducing the stability of *ASB2* and *RARA* [71]. R-2HG can inhibit AML progress by targeting and inhibiting FTO to accumulate m6A on *MYC* and *CEBPA* transcripts and reduce the stability and expression of their mRNAs [72]. Gathering evidence shows the oncogenic function of FTO in various solid tumors, including breast cancer [73] and melanoma [74]. Additionally, the knockdown of FTO increases m6A methylation on pro-tumorigenic genes, including CXCR4 and SOX10, accounting for increased RNA decay via the YTHDF2 reader [74]. Moreover, reducing FTO can enhance the sensitivity for IFNγ and anti-PD-1 blockade [74]. Xu et al. reported that FTO knockdown activated CD8+ T cells faster and killed tumor cells effectively because FTO could inhibit the activation of CD8+ T cells by regulating m6A signals on the bZIP transcription factors family which mediated the expression of glycolytic genes [75]. MA2, an FTO inhibitor, displayed an intrinsic anti-tumor activity with m6A methylation increasing and cell growth inhibited [76, 77]. However, recent studies showed that FTO acts as a tumor suppressor factor. For instance, Fuks found that down-regulated FTO in epithelial cancers such as lung cancer was associated with a worse prognosis, and FTO deficiency augmented the EMT program through increased m6A signals of key mRNAs within the Wnt signaling cascade [78]. Another study showed that FTO suppressed anchorage-independent growth and reduced apoptosis of ICC cells by impairing oncogene *TEAD2* mRNA stability and regulating signaling pathways such as the inflammation pathway and the pyrimidine metabolism pathway [79].

### ALKBH5

Unlike FTO expressing widely, ALKBH5 only shows high expression level in testis. ALKBH5 has been shown to promote cell proliferation and metastasis in glioblastoma stem cells [80]. Recent studies reported that ALKBH5 promoted the progress of AML and the self-renewal of leukemia stem cells rather than normal hematopoietic and blood cells [81]. Up-regulated ALKBH5 in epithelial ovarian cancer impairs autophagy and enhances proliferation and invasion [82]. Conversely, ALKBH5 suppresses the malignancy and metastasis of HCC by destabilizing *LYPD1* mRNA in an IGF2BP1-dependent manner [83].

## Dysregulation of m6A readers in cancer

### YTHDF1

More and more evidence supports that YTHDF1 acts as an oncogene by affecting signaling pathways in cancers, including HCC [84], breast cancer [85], NSCLC [86], CRC [87], and Merkel cell carcinoma [88]. For instance, YTHDF1 affects stem cell-like activity and tumorigenicity of CRC cells by mediating the Wnt/β-catenin pathway and regulating the downstream targets *FZD9* and *WNT6* mRNA [87]. Additionally, oncoprotein c-Myc enhances YTHDF1 expression in colon cancer to induce cancer cell proliferation and resist fluorouracil and oxaliplatin, where YTHDF1 is associated with advanced cancer stages, metastasis, and prognosis [89]. And in NSCLC, the depletion of YTHDF1 will decrease the translation efficiency of m6A-modified transcripts, which inhibits tumor growth under normoxic conditions [86]. And YTHDF1 can recognize and bind to the transcripts marked by m6A that encode lysosomal protease to facilitate antigen degradation, leading to immune escape and incomplete elimination of tumor cells by suppressing the cross-presentation of phagocytic neoantigens and cross-expression of CD8+ T cells [90]. However, in ocular melanoma, YTHDF1, as a tumor suppressor, promotes the translation of *HINT2* mRNA (a tumor suppressor) containing m6A [91].

### YTHDF2

Growing studies support that YTHDF2 not only plays a pro-tumorigenic role but also serves as a suppressor for various cancers. A higher expression level of YTHDF2 has been found in AML [92] and pancreatic cancer [93], indicating its oncogenic role. Notably, YTHDF2 shortens the half-life of m6A-marked TNF receptor 2 (*TNFR2*) mRNA, which usually prevents the accumulation of leukemia cells, thus accelerating the spread of AML [92]. Down-regulation of YTHDF2 significantly restrains cell proliferation and migration. Interestingly, YTHDF2 can promote proliferation and inhibit the EMT process and migration by destroying the stability of *YAP* mRNA, which plays an important role in the Hippo pathway in pancreatic cells [93]. On the other hand, YTHDF2 promotes the degradation of *EGFR* mRNA by directly binding to the m6A modification site at its 3′UTR to inhibit ERK/MAPK signaling pathway, thereby constraining cell proliferation and growth for HCC cells [94]. Recently, several studies showed that YTHDF2 was associated with an inflammation response. For example, in HCC, the decrease of YTHDF2 can cause inflammation and vascular remodeling, promoting tumor progression. Knocking down *YTHDF2* induces a higher abundance of LPS-induced TNF-α, IL-1β, IL-6, and the phosphorylation of NF-κB and MAPK signaling proteins such as p38 and p65 [95].

### YTHDF3

So far, the roles and molecular mechanisms of YTHDF3 have yet to be fully elaborated. YTHDF3 can interact with PABP1 and EIF4G2 to promote *FOXO3* translation and suppress IFN-stimulated genes which are essential for an anti-viral immune response [96]. In addition, YTHDF3 can recognize m6A-labelled lncRNA *GAS5* and mediate its decay to increase YAP expression and promote CRC progression, where *GAS5* serves as a driver for *YAP* nuclear export [97].

### YTHDCs

Due to the lesser exploration, YTHDC1 is an alternative splicing regulator. And differential expressed patterns are drawn for YTHDC1. For example, YTHDC1 is highly expressed in colon adenocarcinoma but not in rectal adenocarcinoma, indicating that the region and even the heterogeneity may cause differences in different functions [98]. In regard to YTHDC2, accumulating evidence supports that YTHDC2 induces tumorigenic effects. YTHDC2 expression level is positively correlated with tumor stages in CRC, indicating that YTHDC2 may promote tumor progression [98]. And YTHDC2 strengthens the translation of *HIF-1α* and *TWIST1* by decoding the m6A modification at 5′UTR to promote EMT and cause tumor metastasis [99].

### IGF2BPs

IGF2BPs can enhance the stability and translation of mRNA under normal and stress conditions, which can lead to the accumulation of carcinogenic products such as MYC [100]. Knockout of IGF2BP in cervical and liver cancer cells can inhibit cell proliferation and invasion [47]. Additionally, IGF2BP1 overexpression can stabilize *PEG10* mRNA with decoding of m6A at 3′UTR to promote endometrial cancer progression [101]. IGF2BP2 can mediate the liver metastasis of colorectal cancer. Mechanistically, YTHDC1 recognizes *circNSUN2* m6A modification and forms a circNSUN2/IGF2BP2/HMGA2 ternary complex in the cytoplasm, which promotes the stability of *HMGA2* mRNA to function on metastasis [102].

Above all, the m6A regulators, including writers, erasers, and readers, are closely related to the initiation, progression, and metastasis of various cancers by regulating specific downstream RNA targets to determine different RNA processes for oncogenes and tumor suppressor genes, including decay, translation, alternative splicing and nuclear export [11, 17, 103]. And the targeted gene may be involved in apoptosis, autophagy, cell cycle, metabolic programming, angiogenesis, EMT, and others by mediating cancer-associated pathways such as the hippo signaling pathway, NFκB, TGFβ, Hedgehog signaling pathway, Notch signaling pathway etc. [50, 104].

## The interplay of m6A and miRNAs in cancers

**Figure 4:**
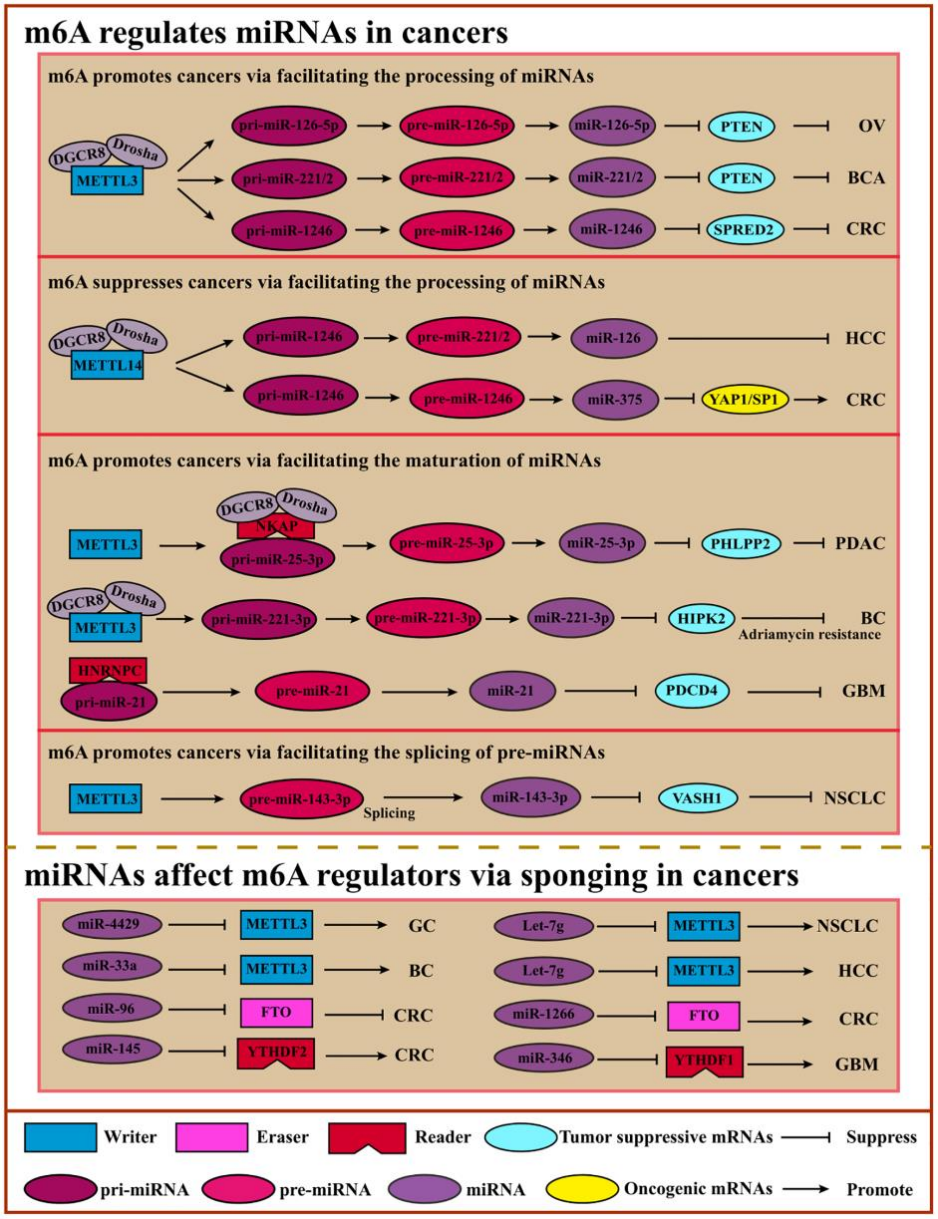
**Crosstalk of m6A and miRNAs.** m6A can modulate miRNA processing, maturating, or pre-miRNA splicing via “Writers” or “Readers” to inhibit or promote cancers. miRNAs regulate m6A regulators by sponging to affect m6A modification, suppressing or promoting cancers. Abbreviations: OV: ovarian cancer; BCA: bladder cancer; CRC: colorectal cancer; HCC: hepatocellular cancer; PDAC: pancreatic ductal adenocarcinoma; BC: breast cancer; GBM: glioblastoma; NSCLC: non-small cell lung cancer.

Usually, miRNAs can regulate gene expression by forming RISC (RNA-induced silencing complex) at the post-transcriptional level with a distributed length of 21–25nt. And miRNAs bind to the 3′UTR of regulated genes, leading to the degradation or translational inhibition of target RNAs. In recent years, growing studies have shown that m6A mediation occurs on miRNAs that affect oncogenes or tumor suppressor genes to impact the tumor process [105, 106] (Figure 4).

M6A methylation affects miRNA processing. For example, miRNA biogenesis involves the following three steps, miRNAs are transcribed as long primary miRNAs (pri-miRNAs) in the nucleus, and these pri-miRNAs are subsequently processed into precursor miRNAs (pre-miRNAs) by a microprocessor complex composed of DGCR8 and DROSHA, and then cleaved by Dicer into mature single-strand miRNAs in the cytoplasm [105].

Intriguingly, the miRNAs biogenesis steps can be affected by m6A regulators in an m6A-dependent manner. METTL3 and HNRNPA2B1 regulate m6A modification on pri-miRNAs to promote the recognition ability of DGCR8 [42, 107]. METTL3 interacts with DGCR8 to promote miR-221/222 maturation which can promote cancer cell proliferation and growth by reducing PTEN expression in bladder cancer cells [108]. In addition, METTL3 also enhances the CRC cells’ metastasis process by facilitating miR-1246 biogenesis and functioning on the miR-1246/*SPRED2/MARK* signaling pathway [109]. Cigarette smoke condensate promotes the growth of pancreatic ductal adenocarcinoma cells by inducing the maturation of METTL3-mediated m6A-modified pri-miR-25-3p [64]. M6A impacts the maturation of pre-miRNAs and the pre-miRNAs to influence cancer progression. For example, miR-143-3p can be cleaved from pre-miR-143-3p under m6A-modified conditions, playing an essential role in NSCLC with brain metastasis by regulating vasohibin-1 (*VASH1*) [110]. Like METTL3, METTL14 can also affect miRNA maturation in an m6A-dependent manner to promote or suppress cancer progression. For instance, METTL14 enhances the identification and binding of DGCR8 to facilitate the maturation of pri-miR-126 whose products miR-126 can inhibit HCC cell migration and invasion [111]. Another study showed that METTL14 suppressed CRC progression by promoting the m6A-dependent maturation of miR-375, which functions on YAP1 and SP1 to suppress proliferation and migration [112]. Moreover, m6A reader HNRNPC induces higher expression of miR-21, leading to lower expression of PDCD4 and active migratory and invasive activity in glioblastoma metastasis [113] (Table 3).

**Table 3:** m6A modulates miRNA processing in cancers.

**m6A regulators**	**Cancer type**	**Mechanism**	**Regulators functions**	**References**
METTL3	Bladder cancer	Promotes the processing of miR-221/222 with DGCR8	↑Proliferation	[108]
METTL3	Colorectal cancer	Promotes the processing of miR-1246 with DGCR8	↑Migration, ↑Invasion	[109]
METTL3	Pancreatic ductal adenocarcinoma	Promotes miR-25-3p maturation	↑Migration, ↑Invasion	[64]
METTL3	Non-small cell lung cancer	Promotes splicing of pre-miR-143-3p	↑Invasion	[110]
METTL3	Ovarian cancer	Promotes miR-126-5p maturation	↑Migration, ↑Invasion	[233]
METTL14	Hepatocellular carcinoma	Promotes the processing of miR-126 with DGCR8	↓Migration, ↓Invasion	[111]
METTL14	Colorectal cancer	Promotes the processing of miR-375 with DGCR8	↓Migration, ↓Growth	[112]
METTL3	Breast cancer	Promotes miR-221-3p maturation	↑Adriamycin resistance	[234]
HNRNPC	Glioblastoma	Promotes miR-21 maturation by binding to pri-miR-21	↑Migration, ↑Invasion	[64]
IGF2BP1	Hepatocellular carcinoma	Impairs the miRNA-directed decay of the SRF mRNA	↑Proliferation	[225]

However, m6A may affect miRNA biogenesis or stability. ALKBH5 interacts with DDX3, which modulates the demethylation of mRNAs by regulating miRNAs [114]. Significantly increased HNONPA2B1 in endocrine-resistant breast cancer reduces the sensitivity to hydro tamoxifen, which is mediated by regulated miRNAs such as miR-29-3p and miR-1268a [115].

Mature miRNAs regulate m6A regulators in cancers. Interestingly, m6A affects the synthesis and function of miRNA directly and impacts the mRNA indirectly mediated by miRNA. IGF2BP2 maintains *RAF-1* mRNA stability by obstructing miRNA-mediated degradation, leading to cancer cell proliferation in CRC [116]. Another study showed that miR-1246 suppresses anti-oncogene *SPRED2* to induce cancer cell migration and invasion. Mechanistically, METTL3 methylated pri-miR-1246 and facilitated the maturation of pri-miR-1246 [109]. In addition to the above, the m6A regulators may be regulated by miRNA. For example, tumor-suppressive miR-34a suppresses potential oncogenic IGF2BP3 to inhibit cell proliferation and invasion [117]. One other study showed that miR-145 negatively regulated YTHDF2 in HCC cells [118]. However, the mechanism of miRNA functioning in m6A modification remains unclear (Table 4).

**Table 4:** miRNA regulates m6A regulators via sponging in cancers.

**miRNA**	**m6A regulators**	**Cancer type**	**miRNA functions**	**References**
miR-4429	METTL3	Gastric cancer	↓Proliferation, ↑Apoptosis	[235]
miR-33a	METTL3	Non-small cell lung cancer	↓Proliferation	[236]
Let-7g	METTL3	Breast cancer	↓Proliferation	[198]
miR-600	METTL3	Non-small cell lung cancer	↓Migration, ↑Apoptosis	[237]
miR-186	METTL3	Hepatocellular carcinoma	↓Migration, ↓Invasion	[238]
miR-96	FTO	Colorectal cancer	↑Migration, ↑Invasion	[239]
miR-1266	FTO	Colorectal cancer	↓Proliferation	[240]
miR-744-5p	HNRNPC	Ovarian cancer	↑Apoptosis	[241]
miR-346	YTHDF1	Glioma	↓Proliferation	[242]
miR-145	YTHDF2	Colorectal cancer/Hepatocellular carcinoma	↓Proliferation	[118]

Collectively, m6A is a critical regulator of miRNA at multiple levels, such as maturation, and reverse regulation for m6A regulators by miRNAs is also vital. Therefore, it is essential to investigate the diverse interactions of m6A and miRNAs directly or indirectly.

## The interplay of m6A and lncRNAs in cancers

**Figure 5:**
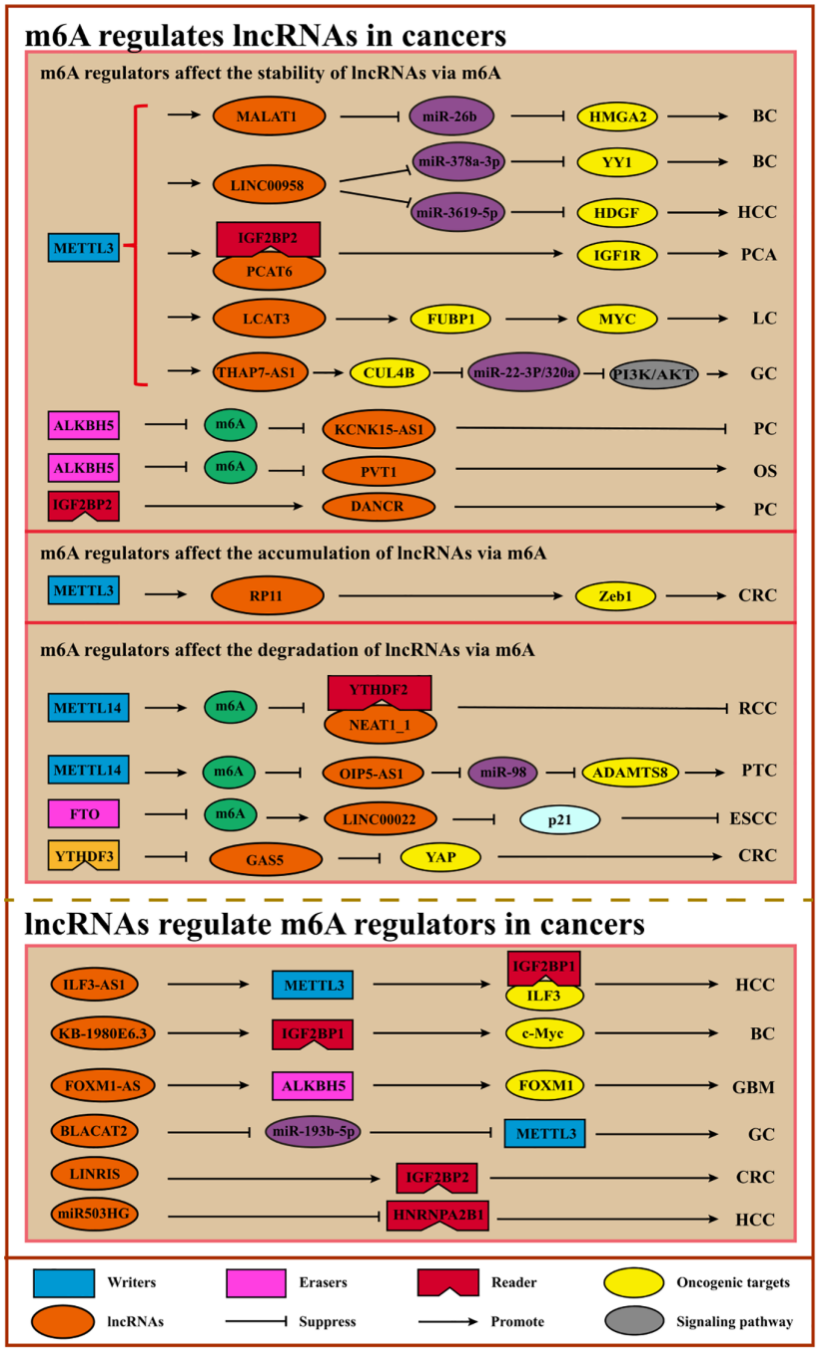
**Interplays of m6A and lncRNAs.** m6A can modulate the stability, degradation, or accumulation of lncRNAs to regulate cancer initiation, progression, and therapy. Conversely, lncRNAs can serve as ceRNAs to regulate m6A regulators via sponging miRNAs or interact directly with m6A regulators to facilitate or inhibit cancer. Abbreviations: BC: breast cancer; HCC: hepatocellular carcinoma; PCA: prostate cancer; LC: lung cancer; GC: gastric cancer; PC: pancreatic cancer; OS: osteosarcoma; CRC: colorectal cancer; RCC: renal cell carcinoma; PTC: papillary thyroid cancer; ESCC: esophageal squamous cell carcinoma; GBM: glioblastoma.

lncRNA, a subgroup of ncRNAs with a length of over 200 nucleotides, can be modified by m6A modification. Researchers are increasingly finding that the interplay of m6A or m6A regulators and lncRNAs could participate in cancer cell proliferation, migration, invasion, or drug resistance. On the one hand, m6A regulators write or recognize the modifiable site lncRNAs which provides to regulate the structure conformation as a structure switch, thereby impacting the RNA-DNA binding ability. On the other hand, lncRNAs serve as endogenous competitive RNAs (ceRNAs) to regulate m6A regulators or directly interact with m6A regulators [119–122] (Figure 5, Tables 5, and 6).

**Table 5:** m6A modification regulates lncRNA in cancers.

**m6A regulators**	**m6A function**	**LncRNA**	**Cancer type**	**Regulator role**	**LncRNA role**	**Mechanism**	**Regulator functions**	**References**
METTL3	writer	MALAT1	Breast cancer	Oncogene	Oncogene	Promotes RNA stability of MALAT1	↑Migration, ↑Invasion	[124]
METTL14	writer	XIST	Colorectal carcinoma	Tumor suppressor gene	Oncogene	Promote degradation of XIST	↓Migration, ↓Invasion	[123]
METTL3	writer	NEAT1	Chronic myeloid leukemia	Oncogene	Oncogene	Induces aberrant expression of NEAT1	↑Migration, ↑Invasion	[243]
ALKBH5	eraser	PVT1	Osteosarcoma	Oncogene	Oncogene	Demethylates PVT1 and enhances its expression	↑Proliferation	[130]
METTL3	writer	LINC00958	Hepatocellular carcinoma/Breast cancer	Oncogene	Oncogene	Promotes the stability of LINC00958	↑Migration, ↑Invasion	[125, 244]
METTL3	writer	FAM225A	Nasopharyngeal carcinogenesis	Oncogene	Oncogene	Promotes the stability of FAM225A	↑Tumorigenesis, ↑Metastasis	[126]
METTL3	writer	RP11	Colorectal carcinoma	Oncogene	Oncogene	Increases the accumulation of RP11	↑Migration, ↑Invasion	[127]
ALHBK5	eraser	KCNK15-AS1	Pancreatic cancer	Tumor suppressor gene	Oncogene	Enhances expression of KCNK15-AS1	↓Migration, ↓Invasion	[131]
FTO	eraser	LINC00022	Esophageal squamous cell carcinoma	Oncogene	Oncogene	Inhibits the decay of LINC00022	↑Proliferation	[132]
IGF2BP2	reader	DANCR	Pancreatic cancer	Oncogene	Oncogene	Promotes the stability of DANCR	↑Proliferation	[133]
METTL3	writer	ABHD11-AS1	Non-small cell lung cancer	Oncogene	Oncogene	Promotes the stability of ABHD11-AS1	↑Proliferation, ↑Warburg effect	[128]
YTHDF3	reader	GAS5	Colorectal carcinoma	Oncogene	Tumor suppressor gene	Promotes the degradation of GAS5	↑Progression	[97]
METTL14	writer	NEAT1	Clear cell renal cell carcinoma	Tumor suppressor gene	Oncogene	Promotes degradation of NEAT1_1	↓Migration, ↓Invasion	[245]
ALKBH5	eraser	NEAT1	Colon cancer, gastric cancer	Oncogene	Oncogene	Promotes demethylation of NEAT1	↑Migration, ↑Proliferation	[129, 246]
METTL3, METTL14	writer	LNCAROD	Headneck squamous cell carcinoma	Oncogene	Oncogene	Promotes the stability of LNCAROD	↑Proliferation	[247]
METTL3	writer	FOXD2-AS1	Cervical cancer	Oncogene	Oncogene	Enhances the stability of FOXD2-AS1	↑Tumorigenesis	[248]
METTL3	writer	THAP7-AS1	Gastric cancer	Oncogene	Oncogene	Enhances the stability of THAP7-AS1	↑Migration, ↑Invasion	[249]
METTL3	writer	MEG3	Hepatocellular carcinoma	Oncogene	Tumor suppressor gene	Promotes degradation of MEG3	↑Migration, ↑Invasion	[250]
METTL3	writer	PCAT6	Prostate cancer	Oncogene	Oncogene	Enhances the stability of PCAT6	↑Metastasis	[251]
METTL3	writer	LCAT3	Lung cancer	Oncogene	Oncogene	Enhances the stability of LCAT3	↑Migration, ↑Invasion	[252]
METTL14	writer	Lnc-LSG1	Clear cell renal cell carcinoma	Tumor suppressor gene	Oncogene	Blocks the interaction of Lnc-LSG1 and ESPR2	↓Migration, ↓Invasion	[253]
METTL14	writer	OIP5-AS1	Papillary thyroid cancer	Oncogene	Tumor suppressor gene	Inhibits OIP5-AS1 expression	↑Migration, ↑Invasion	[254]

**Table 6:** LncRNA modulates m6A regulators in cancer.

**LncRNA**	**m6A regulators**	**Type**	**Cancer type**	**LncRNA role**	**Regulator role**	**Mechanism**	**References**
LINC00470	METTL3	writer	Gastric cancer	Oncogene	Oncogene	LINC00470 promotes METTL3-mediated *PTEN* mRNA degradation	[136]
LINC00942	METTL14	writer	Breast cancer	Oncogene	Oncogene	Recruits METTL14 to mediate CXCR4 and *CYP1B1* mRNA stability.	[255]
GATA3-AS	KIAA1429	writer	Hepatocellular carcinoma	Oncogene	Oncogene	Mediate interaction of KIAA1429 with *GATA3* pre-mRNA to methylate and degrade *GATA3* pre-mRNA	[70]
UTM2A-AS1	METTL3	writer	Lung adenocarcinoma	Oncogene	Oncogene	Regulates the miR-590-5p/METTL3 axis	[256]
LINC00240	METTL3	writer	Gastric cancer	Oncogene	Oncogene	Modulates miR-338-5p/METTL3 axis	[257]
BLACAT2	METTL3	writer	Gastric cancer	Oncogene	Oncogene	Modulates miR-193b-5p/METTL3	[258]
IFL3-AS1	METTL3	writer	Hepatocellular carcinoma	Oncogene	Oncogene	Inhibits the degradation of *ILF3* mRNA	[259]
FOXM1-AS	ALKBH5	eraser	Glioblastoma	Oncogene	Oncogene	Promotes the interaction of ALKBH5 with FOXM1 to enhance demethylation of FOXM1	[80]
GAS5-AS1	ALKBH5	eraser	Cervical cancer	Tumor suppressor gene	Tumor suppressor gene	Interacts with ALKBH5 to demethylate *GAS5* to promote tumor suppressor gene *GAS5* stability	[260]
MALAT1	IGF2BP2	reader	Thyroid cancer	Oncogene	Oncogene	miR-204/IGF2BP2/m6A-MYC	[134]
LINRIS	IGF2BP2	reader	Colorectal cancer	Oncogene	Oncogene	LINRIS maintains the stability of IGF2BP2	[135]
KB-1980E6.3	IGF2BP1	reader	Breast cancer stem cells	Oncogene	Oncogene	Recruits IGF2BP1 to mediate *c-Myc* mRNA stability.	[137]
LINC00857	YTHDC1	reader	Colorectal cancer	Oncogene	Oncogene	Interacts with YTHDC1 to stabilize SLC7A5	[261]
LIN28B-AS1	IGF2BP1	reader	Hepatocellular carcinoma, lung adenocarcinoma	Oncogene	Oncogene	Activate LIN28B by interacting with IGF2BP1	[262]
miR503HG	HNRNPA2B1	reader	Hepatocellular carcinoma	Tumor suppressor gene	Oncogene	Promotes HNRNPA2B1 degradation	[263]
DMDRMR	IGF2BP3	reader	Clear cell renal cell carcinoma	Oncogene	Oncogene	Up-regulates m6A-modified CDK4 via interacting with IGF2BP3.	[264]
LINC01234	HNRNPA2B1	reader	Non-small-cell lung cancer	Oncogene	Oncogene	Facilitates the processing of pri-miR-106b	[265]

M6A regulates lncRNA to promote or suppress cancer progression. It has been shown that METTL3, METTL14, and RBM15 can mediate the silencing of lncRNA XIST in a YTHDF2-dependent m6A manner [22, 123]. As for oncogenes, one study showed that METTL3 up-regulated *MALAT1* expression to facilitate breast cancer cell migration and invasion via the *MALAT1*/miR-26b/*HMGA2* axis [124]. Moreover, m6A modification on *LINC00958* mediated by METTL3 induces its higher expression with its RNA transcripts stabilizing, leading to HDGF up-regulation by sponging miR-3619-59 in HCC [125]. In nasopharyngeal carcinogenesis, METTL3 stabilized *FAM225A* via m6A modification and up-regulated *FAM225A* expression, thereby contributing to tumorigenesis and metastasis by sponging miR-590-3p/miR-1275 and up-regulating *ITGB3* with the FAK/PI3K/AKT signaling pathway being activated [126]. METTL3 also increases *RP11* accumulation via m6A modification in CRC, thus, preventing the degradation of *ZEB1* by accelerating the degradation of E3 ligases SIAH1 and FBXO45 [127]. *ABHD11-AS1*, whose stability can be strengthened by METTL3, promotes NSCLC cell proliferation and the Warburg effect [128]. In addition to the writers, the m6A erasers and readers also regulated lncRNAs. For instance, overexpression of ALHBK5 in GC cells promotes invasion and metastasis by inhibiting *NEAT1* methylation [129]. ALHBK5-mediated demethylation of lncRNA *PVT1* promotes osteosarcoma cell proliferation [130]. ALHBK5 can also serve as a tumor suppressor. ALHBK5-mediated m6A demethylation on lncRNA *KCNK15-AS1* can up-regulate *KCNK15-AS1* expression to suppress pancreatic cancer cell migration and invasion while facilitating apoptosis [131]. Another eraser FTO inhibits the *LINC00022* decay by decreasing its m6A modification, and *LINC00022* promotes ESCC cell proliferation and cell-cycle progression by binding to p21 and promoting its degradation [132]. Regarding readers, IGF2BP2 stabilizes DANCR by recognizing DANCR modification, leading to the proliferation of pancreatic cancer cells [133] (Table 5).

Conversely, lncRNAs can affect m6A via regulating m6A regulators directly or indirectly. For example, MALAT1, considered an oncogene, acts as a ceRNA competitively binding to miR-204 to up-regulate IGF2BP2 expression and enhance MYC expression by m6A modification in thyroid cancer [134]. Moreover, LINRIS maintains the stability of IGF2BP2 by blocking ubiquitination, leading to CRC cell proliferation via MYC-mediated glycolysis [135]. Another study showed that LINC00470 interacted with METTL3 to facilitate *PTEN* mRNA degradation, thereby accelerating distal migration [136]. LncRNA *KB-1980E6.3* is overexpressed and promotes breast cancer stem cell tumorigenesis and self-renewal via recruiting IGFBP1 and retaining *c-Myc* stability [137]. Furthermore, YTHDF3 accelerates the degradation of GAS5 to regulate YAP signaling and promote CRC progression [97]. Additionally, lncRNA *ARHGAP5-AS1* can recruit METTL3 to stimulate *ARHGAP5* mRNA m6A modification and stabilize *ARHGAP5* mRNA, leading to chemoresistance in gastric cancer [138] (Table 6).

Overall, the interactions between m6A modification or m6A regulators and lncRNAs provide insight for a better understanding of cancer initiation, progression, and therapy.

## The interplay of m6A and circRNAs in cancers

**Figure 6:**
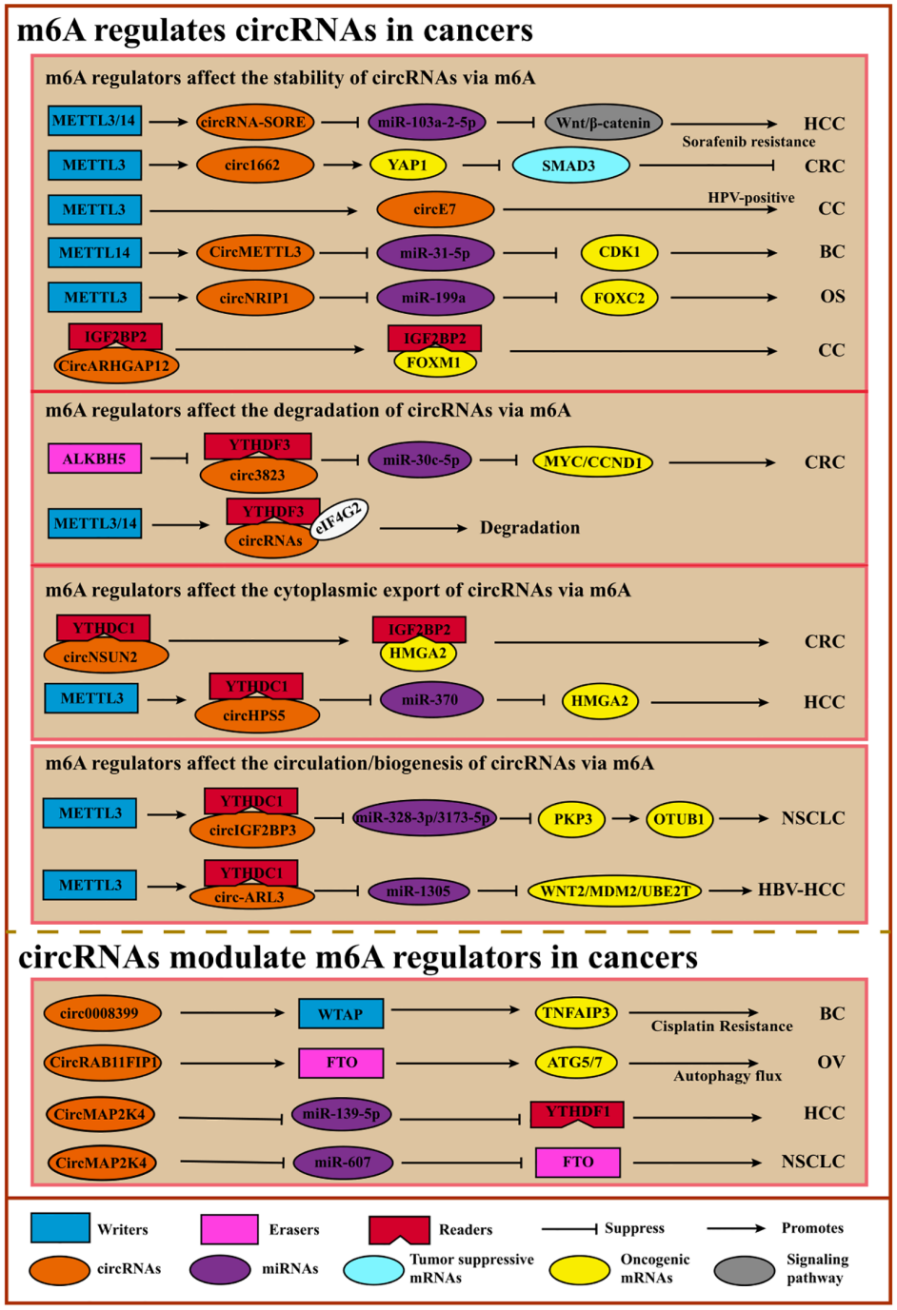
**Crosstalk between m6A and circRNAs.** m6A facilitates the circulation, accumulation, translocation, translation, or degradation of circRNA to promote or suppress cancers via diverse cancer-associated signaling pathways or tumor-related oncogene/suppressors. On the contrary, circRNAs regulate m6A regulators like lncRNAs.

Circular RNAs, a group of ncRNAs characterized by covalently closed loop structures with neither 5′ to 3′ polarity nor polyadenylated tail, are more stable than linear RNAs due to their ring-closed structure [139]. Accumulating evidence shows the crosstalk between m6A modification or m6A regulators and circRNAs. Indeed, M6A modification modulates diverse functions of circRNAs, including expression, translation, location, and interaction with RNA or protein. Conversely, circRNAs can also affect m6A modification by regulating the m6A regulators, including writers, erasers, or readers. Surprisingly, aberrant circRNAs have been found and modified by m6A in diverse cancers. Exploring the crosstalk of m6A and circRNA is necessary for cancer therapy [119, 140] (Figure 6, Table 7).

**Table 7:** The functions of m6A-modified circRNAs in cancers.

**m6A regulators**	**m6A function**	**circRNA**	**Cancer type**	**Regulator role**	**circRNA role**	**Mechanism mediated by m6A**	**circRNA Functions**	**References**
METTL3	writer	circNRIP1	Osteosarcoma	oncogene	oncogene	Promotes circNRIP1 expression	↑Proliferation, ↑Migration	[141]
ALKBH5/YTHDF3	eraser/reader	circ3823	Colorectal cancer	tumor suppressor gene	oncogene	Reduces circ3823 stability	↑Proliferation, ↑Metastasis, ↑Angiogenesis	[142]
METTL14/FTO	writer/eraser	circMETTL3	Breast cancer	oncogene/tumor suppressor gene	oncogene	Promotes/Reduces circMETTL3 expression	↑Proliferation, ↑Migration, ↑Invasion	[143]
YTHDC1	reader	circNSUN2	Colorectal cancer	oncogene	oncogene	Facilitates circNSUN2 cytoplasmic export and mediates HMGA2 stabilization	↑Metastasis	[102]
METTL3/YTHDC1	writer/reader	circHPS5	Hepatocellular carcinoma	oncogene	oncogene	Facilitates m6A-modified circHPS5 accumulation and cytoplasmic export	↑Proliferation, ↑Migration	[148]
METTL3/14	writer	circRNA-SORE	Hepatocellular carcinoma		sustains sorafenib resistance	Increases circRNA-SORE stability	Sustains sorafenib resistance	[266]
METTL3/YTHDC1	writer/reader	circ-ARL3	HBV- Hepatocellular carcinoma	oncogene	oncogene	Favors reverse splicing and biogenesis of circ-ARL3	↑Tumorigenesis	[149]
YTHDF2	reader	circASK1	lung adenocarcinoma		ameliorates gefitinib resistance	Increases endoribonucleolytic cleavage of m6A-modified circASK1	Ameliorate gefitinib resistance, ↑Apoptosis	[267]
METTL3/YTHDC1	writer/reader	circIGF2BP3	Non-small-cell lung cancer	oncogene	oncogene	Promotes circIGF2BP3 circularization	Facilitate tumor immune evasion	[150]
IGF2BP2	reader	circARHGAP12	cervical cancer		oncogene	Promotes circARHGAP12 stability	↑Progression	[144]
		circPVRL3	Gastric cancer		tumor suppressor gene	Promotes the circPVRL3 translation	↓Proliferation, ↓Migration	[151]
METTL3	writer	CircE7	cervical carcinoma		oncogene	Promotes CircE7 stability	↑Growth	[145]
METTL3	writer	circ1662	Colorectal cancer	oncogene	oncogene	Induces circ1662 expression and install m6A on it	↑Migration, ↑Invasion	[146]
METTL3	writer	circCUX1	Hypopharyngeal squamous cell carcinoma			Promotes circCUX1 stability and expression	Confers radio-resistance	[268]

m6A affects the location, biogenesis, stabilization, or translation of circRNAs, leading to cancer proliferation, migration, or invasion [140]. For example, in osteosarcoma, METTL3-mediated m6A modification on circNRIP1 can promote its expression to enhance cell proliferation and migration [141]. M6A-modified *Circ3823* contributes to CRC cell proliferation and metastasis, which is regulated negatively by ALKBH5 and YTHDF3 [142]. Another study showed a similar result: high expression of *circMETTL3* induced by METTL14-mediated m6A modification leads to breast cancer cell migration and invasion [143]. Other studies also reported that m6A could stabilize the circ-RNAs to impact cancer progressions, such as *circARHGAP12* and *CircE7* in cervical cancer [144, 145] and *circ1662* in CRC [146]. As mentioned above, some studies showed that m6A regulated the translocation of circRNAs. In colorectal cancer, m6A mediation facilitates *circNSUN2* cytoplasmic export and further mediates *HMGA* stabilization to promote cancer metastasis [147]. Another study reported that METTL3 could direct circHPS5 formation and METTL3-controlled m6A could also facilitate the circHPS5 accumulation and cytoplasmic export, indicating that m6A regulators may participate in the formation of circRNAs [148]. Oncogenic *circ-ARL3*, whose biogenesis is controlled by m6A modification, promotes tumorigenesis in HBV-HCC [149]. Furthermore, METTL3-mediated m6A modification promotes *circIGF2BP3* circularization, facilitating NSCLC cells’ immune evasion. Although circRNAs are considered ncRNAs, some circRNAs have the potential to encode short peptides or proteins [150]. M6A promotes *circPVRL3* translation, and *circPVRL3* can inhibit GC cell migration and invasion [151]. Despite these initial investigations, the mechanisms underlying the functions of m6A-modified circRNAs still need to be explored, especially the translation process (Table 7).

circRNAs can regulate m6A modification on specific target genes by affecting the m6A regulator’s expression or functions. For instance, *circNOTCH1* can competitively bind to METTL14 to regulate NOTCH1 mRNA modification, thereby controlling *NOTCH1* expression [152]. CircRNAs can also interact with writers or erasers to control target gene m6A modification to further regulate the expression. *CircARHGAP12* can interact with IGF2BP2 to accelerate the stability of *FOXM1* mRNA, leading to cervical cancer progression [144]. Another study revealed that *CircRAB11FIP1* promoted epithelial ovarian cancer cell proliferation and invasion via interacting with FTO to decrease the expression of *ATG5* and *ATG7* by demethylating their m6A modification [153]. And other circRNAs like *circARHGAP12* in cervical cancer [144] and *circ0008399* in bladder cancer [154] also perform similar functions. Like lncRNA, circRNAs can also act as ceRNAs to regulate the m6A regulators. For example, *CircMAP2K4* and hsa_circ_0072309 enhance HCC and NSCLC cells proliferation and migration via the *CircMAP2K4*/miR-139-5p/*YTHDF1* axis [155] and *hsa_circ_0072309*/miR-607/*FTO* axis [156], respectively. Importantly, circRNAs can only regulate m6A modification indirectly via m6A regulators rather than themselves directly (Table 8).

**Table 8:** CircRNA modulates m6A regulators in cancer.

**circRNA**	**m6A regulators**	**m6A function**	**Cancer type**	**circRNA role**	**Mechanism**	**circRNA Functions**	**References**
circARHGAP12	IGF2BP2	reader	cervical cancer	oncogene	Interacts with IGF2BP2 to accelerates the stability of FOXM1 mRNA	↑Progression	[144]
CircRAB11FIP1	FTO	eraser	epithelial ovarian cancer	oncogene	Interacts with FTO to decrease expression of ATG5 and ATG7	↑Proliferation, ↑Invasion	[153]
circ_KIAA1429	YTHDF3	reader	Hepatocellular carcinoma	oncogene	YTHDF3 enhanced Zeb1 mRNA stability	↑Proliferation, ↑Migration, ↑Invasion	[269]
CircMAP2K4	YTHDF1	reader	Hepatocellular carcinoma	oncogene	CircMAP2K4/miR-139-5p/YTHDF1 axis	↑Proliferation	[155]
circ0008399	WTAP	writer	bladder cancer	oncogene	Interacts with WTAP and increase TNFAIP3 stability	Reduce chemosensitivity to CDDP	[154]
circNOTCH1	METTL14	writer	Non-small-cell lung cancer	oncogene	Regulates NOTCH1 mRNA m6A modification by competing for METTL14 binding	↑Growth	[152]
hsa_circ_0072309	FTO	eraser	Non-small-cell lung cancer	oncogene	hsa_circ_0072309/miR-607/FTO axis	↑Tumorigenesis, ↑Invasion	[156]
CircMEG3	METTL3	writer	liver cancer	tumor suppressor gene	Inhibits the expression of METTL3 dependent on HULC	↓Growth	[270]
circNDUFB2	IGF2BPs	reader	Non-small-cell lung cancer	tumor suppressor gene	Destabilizes IGFBPs	↓Growth	[271]
CircPTPRA	IGF2BP1	reader	Bladder cancer	tumor suppressor gene	Blocks the recognition of m6A via interacting with IGF2BP1	↓Proliferation, ↓Migration, ↓Invasion	[272]

## m6A databases and m6A sites predictors

To explore the function of m6A systematically and conveniently, the investigator collected and built diverse databases such as m6A-Atlas [157], m6A2Target [158], RMVar [159], and RMDisease [160]. Among them, some summarize the existing m6A modification sites and the potential targets of m6A regulators, such as m6A-Atlas, MET-DB v2.0 [161], RMBase v2.0 [162], and m6A2Target [158]. While some research teams provide the associations between m6A modification sites or m6A-associated variants, m6A-associated histone modification, and chromatin accessibility regions and diseases such as REPIC [163] and RMDisease [160]. And RMDisease summarizes disease-specific genetic variants that affect RNA modification sites (Table 9).

**Table 9:** m6A-related databases.

**Database**	**Year**	**Characteristics**	**Species**	**URL**	**Reference**
RMBase v2.0	2018	It is a update version with modules “Motif”, “modRBP” and “modMetagene” for visualization of RNA modification sites and explore their potential functions such as SNP or RBP regions.	Human/Mouse/Yeast/Arabidopsis/Zebrafish/Rat/Macaque/Chimpanzee/E.coli/Fruit fly/P.aeruginosa/ Pig/Rhesus	http://rna.sysu.edu.cn/rmbase/	[162]
M6A2Target	2020	It provides the potential targets or validated targets for m6A writers, erasers and readers.	Human/Mouse	http://m6a2target.canceromics.org/	[158]
m6A-Atlas	2020	It provides a high-confidence collection of m6A modification sites and the quantitative epitranscriptome profiles	Human/Mouse/Rat/Zebrafish/Fly/Arabidopsis/Yeast	http://www.xjtlu.edu.cn/biologicalsciences/atlas	[157]
RMVar	2021	It provides RNA modification (RM)-associated variants for 9 kinds of RNA modifications and their potential function on posttranscriptional regulation and disease.	Human/Mouse	https://rmvar.renlab.org/	[159]
m6AVar	2018	It provides m6A modification sites and potential function of m6A associated variants on post-transcriptional regulation and disease.	Human/Mouse	http://m6avar.renlab.org/	[273]
REPIC	2020	It provides m6A modification sites, m6A-associated histone modification and chromatin accessibility regions	Human/Mouse/Yeast/Arabidopsis/Zebrafish/Rat/Macaque/Chimpanzee/E.coli/Fruit fly/P.aeruginosa	https://repicmod.uchicago.edu/repic	[163]
MET-DB v2.0	2018	It provides context specific m6A peaks as well as m6A sites at single-base level and the targets of m6A regulators.	Human/Mouse/Rat/Zebrafish/Fly	http://compgenomics.utsa.edu/MeTDB; http://www.xjtlu.edu.cn/metdb2	[161]
CVm6A	2019	It provides a visualization interface for m6A patterns in cell lines and their potential function in disease and development.	Human/Mouse	http://gb.whu.edu.cn:8080/CVm6A	[274]
m6Acomet	2019	It provides gene ontology functions associated m6A sites.	Human	http://www.xjtlu.edu.cn/biologicalsciences/m6acomet	[275]
M6ADD	2021	It provides analysis interface for m6A modification and the associations between m6A modification and gene disorders and diseases	Human/Mouse	http://m6add.edbc.org/	[276]
RMDisease	2021	It provides disease specific genetic variants that affect RNA modifications sites.	Human	http://www.xjtlu.edu.cn/biologicalsciences/rmd	[160]
RNAmod	2019	It is a web platform for mRNA modifications analysis, annotation and visualization, including m6A distribution, modification coverage and functional ontology.	Human/Mouse/Yeast/Arabidopsis/Zebrafish/Rat/Macaque/Chimpanzee/Fruit fly/Pig	https://bioinformatics.sc.cn/RNAmod	[277]
DRUM	2019	It is a multi-layer heterogeneous network-based approach by utilizing RWR algorithm to integrate the associations among genes, m6A sites and diseases.	Human/Mouse	http://www.xjtlu.edu.cn/biologicalsciences/drum	[278]
RNAWRE	2020	It deposits RNA modification effectors	/	http://rnawre.bio2db.com/	[279]

Additionally, many tools and platforms predict m6A modification sites based on m6A-seq or MeRIP-seq [164] datasets across diverse species. Generally, the predictors for m6A sites go through the following steps:

1. Transforming the RNA sequences with/without m6A sites into feature representations, including PseDNC, PseTNC, NCP, ANF, DNC, PSNP, PSDP, NC, PCP, AC, CC, NPPS, STTNC, PSNSP, PSDSP, KSNPF, PS(k-mer) NP, PCPs and RFHC.
2. Selecting essential features using IFS, RFE, or SFS (optional step).
3. Training the classifier based on the selected features to generate the model, such as random forest model, SVM, KNN, or deep neural network.
4. Voting with ensemble classifier (optional step).
5. Evaluating the performance of the classifiers using a general evaluation index such as ROC.

Notably, it is necessary to establish an evaluation system to evaluate the merits of these software for better supervision and efficiently convenient for users because of too many prediction tools (Table 10).

**Table 10:** Computational methods for predicting m6A modification from RNA sequences.

**Predictor**	**Year**	**Characteristics**	**Species**	**URL**	**Reference**
SRAMP	2016	It integrates three random forest classifiers to predict m6A modification sites.	/	http://www.cuilab.cn/sramp/	[280]
m6AViewer	2017	It is a tools to detect, analyze and visualize the m6A peaks with sequencing dataset.	/	http://dna2.leeds.ac.uk/m6a	[281]
RNAMethPre	2016	It integrates multiple features of mRNA and provides a trained SVM classifier to predict m6A sites	Human/Mouse		[282]
WHISTLE	2019	It is webserver for m6A site prediction and investigation of its potential biological process.	Human/Mouse/Yeast/	http://whistle-epitranscriptome.com;www.xjtlu.edu.cn/biologicalsciences/whistle	[283]
AthMethPre	2016	It adopted SVM classifier to predict mRNA m6A modification sites.	Arabidopsis thaliana	http://bioinfo.tsinghua.edu.cn/AthMethPre/	[284]
RFAthM6A	2018	It provides four independent models called RFPSNSP, RFPSDSP, RFKSNPF and RFKNF to predict m6A sites	Arabidopsis thaliana	https://github.com/nongdaxiaofeng/RFAthM6A	[285]
M6A-BiNP	2021	It provides two feature-encoding methods called PSP-PMI or PSP-PJMI to predict m6A sites	Human/Mouse/Rat	https://github.com/Mingzhao2017/M6A-BiNP	[286]
M6AMRFS	2018	It employs XGBoost classifier trained on dinucleotide binary encoding and Local position-specific dinucleotide frequency for m6A sites prediction	Human/Mouse		[287]
DNN-m6A	2021	It provides DNN-based m6A prediction by adopting different strategies like TNC, ENAC and PSNP to extract sequence features and selecting feature subsets via elastic net	Human/Mouse/Rat	http://github.com/GD818/DNN-m6A	[288]
m6A-Maize	2021	It is a supervised learning model to predict m6A sites and provides a collection of traits-associated m6A mutations.	Maize	http://www.xjtlu.edu.cn/biologicalsciences/maize	[289]
TS-m6A-DL	2021	It is a predictor based on DNN trained by sequences with one-hot-encoding method	Human/Mouse/Rat	http://nsclbio.jbnu.ac.kr/tools/TS-m6A-DL/	[290]
iRNA-m6A	2020	It adopted SVM classifier based on selected feature by mRMR to predict mRNA m6A modification sites.	Human/Mouse/Rat	http://lin-group.cn/server/iRNA-m6A	[291]
m6AGE	2021	It utilizes CatBoost model to predict m6A sites based on sequence-derived and graph embedding features	S. cerevisiae/ A.thaliana/ Human	https://github.com/bokunoBike/m6AGE	[292]
M6A-GSMS	2021	It employs GBDT to select features and train tracking model by combining seven basic classifiers.	A.thaliana/D.melanogaster/M.musculus/ S.cerevisiae/ Human	https://github.com/Wang-Jinyue/M6A-GSMS	[293]
iRNA-3typeA	2018	It is a SVM predictor based on PseKNC for m6A, m1A and A-to-I prediction.	/	http://lin-group.cn/server/iRNA-3typeA/	[294]
iRNA-Methyl	2015	It is a predictor for m6A site based on sequence editing via pseudo nucleotide composition.	/		[295]
BERMP	2018	It is a deep-learning classifier with bidirectional Gated Recurrent Unit (BGRU) for m6A prediction across multiple species.	Human/Mouse/Arabidopsis/Yeast		[296]
M6APred-EL	2018	It is an ensemble of three SVM based on PS(k-mer) NP, PCPs and RFHC for m6A prediction.	/		[297]
M6ATH	2016	It is a SVM-based predictor for identifying m6A sites in A.thaliana.	A.thaliana		[298]
TargetM6A	2016	It is a SVM-based predictor trained by IFS-selected features that consist of PSNP/PSDP and NC transformed from RNA sequences.	/	http://csbio.njust.edu.cn/bioinf/TargetM6A	[299]
pRNAm-PC	2016	It is a predictor for m6A site based on sequence editing via pseudo nucleotide composition.	/		[300]
M6A-HPCS	2016	It is an m6A site predictor based on HPCS algorithm under sequence feature representations including PseDNC, AC and CC.	S. cerevisiae (Example)	http://csbio.njust.edu.cn/bioinf/M6A-HPCS	[301]
RAM-ESVM	2017	It is an ensemble SVM classifiers trained by PseDNC, Motif features and gapped K-mers for m6A sites prediction in S. cerevisiae.	S. cerevisiae		[302]
iRNA-PseColl	2017	It is a SVM-based platform to identify the occurrence sites for RNA modifications based on PseKNC.	/		[303]
RAM-NPPS	2017	It is a SVM-based predictor for m6A identification rapidly based on a novel feature representation algorithm called multi-interval nucleotide pair position specificity.	Human/Arabidopsis/Yeast		[304]
iMethyl-STTNC	2018	It is proposed for identification of m6A sites and is built on SVM, probabilistic neural network and KNN with feature extractions strategies, including PseDNC, PseTNC, STTNC and STNC.			[305]
Gene2Vec	2019	It is a soft vote predictor for m6A sites by integrating four different deep learning classifiers with shifting window.	Human/Mouse		[306]
iRNA(m6A)-PseDNC	2018	It is a predictor for m6A site based on sequence editing via pseudo nucleotide composition.	S. cerevisiae	http://lin-group.cn/server/iRNA(m6A)-PseDNC.php	[307]
ConsRM	2021	It is a scoring framework that embedded with phastCons and phyloP to distinguish conserved m6A sites.	Human/Mouse/Fly/Zebrafish	https://www.xjtlu.edu.cn/biologicalsciences/con	[308]
Funm6AViewer	2021	It examines the distribution of m6A sites, prioritizes genes mediated by m6A modification and functionally gene regulatory network	/	https://www.xjtlu.edu.cn/biologicalsciences/funm6aviewer;https://github.com/NWPU-903PR/Funm6AViewer	[309]
xPore	2021	It identifies the m6A sites from nanopore direct RNA sequencing	Human/Mouse	https://github.com/GoekeLab/xpore	[310]
MASS	2021	It is a multi-task computational framework to capture m6A features and their regulatory functions across multiple species.	Human/Mouse/Yeast/ Arabidopsis/ Zebrafish/Rat/ Macaque/ Chimpanzee/ Fruit fly/Pig	https://github.com/mlcb-thu/MASS	[311]

## Methods detecting m6A sites

m6A modification sites can be detected directly by nanopore sequencing such as xPore and identified by isolating modified RNA fragments based on immunoprecipitation or specific RNA chemistry and combining them with sequencing [165, 166] (Table 11).

**Table 11:** Experimental methods for detecting m6A modification.

**Methods**	**Year**	**Characteristics**	**References**
MeRIP-seq	2015	Combine m6A methylated mRNA fragments and high-throughput sequencing to detect m6A modification regions about 100nt-200nt. Cannot reflect the precise positions of m6A sites.	[167]
PA-m6A-seq	2015	Improve the resolution to 30nt around m6A modification sites via 4SU strengthening crosslinks. Can only detect m6A modification sites around 4SU operating sites with a resolution of 20nt-30nt.	[168]
miCLIP-seq	2015	Combine immunoprecipitation and high-throughput sequencing to detect m6A residues precisely at single-base resolution.	[169]
m6A-REF-seq	2019	Detect m6A sites in the whole transcriptome in an antibody-independent manner.	[170]
DART-seq	2019	Induce C-to-U deamination at sites adjacent to m6A residues to detect m6A.	[171]
m6A-label-seq	2020	Detect the m6A sites with a single-base resolution by marking the resources of methylation sites	[172]
m6A-SEAL	2020	Utilize streptavidin beads to enrich biotin-labeled m6A-modified sites for sequencing analysis.	[173]
m6A-seq2	2021	Enhance robust m6A quantification at the site, gene, and sample resolution via multiple m6A immunoprecipitation techniques with bar code and mixed samples.	[174]
miCLIP2	2021	Use low-input material to construct high-complexity libraries. Identify reliable m6A sites regardless of false positives induced by broad antibody reactivity, in combination with machine learning.	[175]
M6A-LAIC-seq	2016	Quantitatively detect m6A presence for genes or isoforms on a transcriptome-wide level based on the spike-in External RNA Controls Consortium.	[176]
SCARLET	2014	A digestion-based method to determine the percentage of m6A with single-nucleotide resolution for mRNAs and lncRNAs. It is not yet feasible for high-throughput applications.	[178]
SELECT	2018	Precisely distinguish m6A modification residues at a single base resolution. Does not require antibody IP or isotope labeling, thus reducing cost and can be widely used.	[177]
Nanopore direct-RNA sequencing	2021	Nanocompore can detect different RNA modifications with position accuracy *in vitro*.	[312]
HPLC-coupled mass spectrometry	2014	Short analysis time, good resolution and high sensitivity.	[313, 180]
Colorimetry	2016	Easily detect the overall level of RNA m6A modification.	[314]
Dot blot	2017	Dot blot analysis of RNA m6A is relatively easy, rapid and cost-effective.	[71]
PAR-CLIP	2010	It is based on incorporation of photoactivatable nucleoside analogs into nascent RNA.	[181]
Electrochemical immunosensor	2015	It showed a linear range for methylated RNA from 0.01 to 10 nM and the detection limit was 2.57 pM.	[182]

A developed method called methylated RNA immunoprecipitation sequencing (MeRIP-seq) can combine m6A methylated mRNA fragments and high-throughput sequencing to detect m6A modification regions at about 100nt-200nt [167]. However, this method is complicated due to the largely overlapped reads for the similar m6A-containing fragments, thereby leading to prominent peaks spanning m6A residues, which indicates that MeRIP-seq method needs to reflect the precise positions of m6A sites. Then, one technique called PA-m6A-seq can improve the resolution to 30nt around m6A modification sites via 4SU strengthening crosslinks. However, PA-m6A-seq can only detect m6A modification sites around 4SU operating sites with a resolution of 20nt-30nt [168]. Another technology called miCLIP (m6A individual-nucleotide-resolution cross-linking and immunoprecipitation) can detect m6A residues precisely and overcome the disadvantages of PA-m6A-seq. Additionally, miCLIP-seq combines immunoprecipitation and high-throughput sequencing to identify m6A residues at high resolution, leading to the detection of m6A on snoRNA with high probability [169]. While m6A-REF-seq can quantify the m6A levels at single-base resolution and accurately detect m6A sites in the whole transcriptome in an antibody-independent manner, which is achieved by newly discovered RNA endonuclease MazF that is highly sensitive to m6A [170]. Another antibody-free method called DART-seq utilizes the fusion constructed by APOBEC1 (cytidine deaminase) and the YTH domain to induce C-to-U deamination at sites adjacent to m6A residues, thereby detecting m6A sites [171]. A recently developed method, termed m6A-label-seq, directly detects the m6A sites with a single-base resolution by marking the resources of methylation sites [172]. Briefly, METTL3/METTL14 utilize allyl-SAM or allyl-SeAM to install the methylation on RNA sites to obtain a6A, which can induce cyc-A formation under mild iodine conditions; hence m6A sites are identified via the mutations resulting from base mismatch caused by reverse transcribing from cyc-A to cDNA. At the same period, another technology named m6A-SEAL enables m6A sites to label biotin by methanethiosulfonate reacting with free sulfhydryl group on dm6A generated via m6A/hm6A/dm6A steps and utilizes streptavidin beads to enrich biotin-labeled m6A-modified sites for further sequencing analysis [173]. m6A-seq2, a newly updated method from m6A-seq, enhances robust m6A quantification at the site, gene, and sample resolution via multiple m6A immunoprecipitation techniques with bar codes and mixed samples [174]. Another updated technology, miCLIP2, characteristics of high-complexity libraries from less input material, identifies reliable m6A sites regardless of false positives induced by broad antibody reactivity, combined with machine learning [175].

The m6A-LAIC-seq method can quantify m6A presence for genes or isoforms on a transcriptome-wide level based on the spike-in External RNA Controls Consortium [176]. SELECT [177], MeRIP-qPCR [167] and MazF-qPCR [170] are often used to detect and quantify the m6A-containing fragments or genes. Method SCARLET can precisely distinguish m6A modification residues at single base resolution for mRNAs and lncRNAs based on specific RNase H, radiolabeling, and TLC [178]. And SCARLET can also be applied to detect m1A and Nm RNA modification sites. However, SCARLET is challenging to being widely used due to its complicated step and a significant dose of a radioactive isotope. And SCARLET can also be applied to detect m1A and Nm RNA modification sites. Single-base elongation and ligation-based qPCR amplification method (SELECT) is used for detecting m6A single base resolution in low abundance transcripts [177]. SELECT is based on two characteristics of m6A: (i) it blocks the single base extension of DNA polymerase during the reverse transcription process; (ii) reduces the nick ligation efficiency of ligases. This method does not require antibody IP or isotope labeling, thus reducing cost, and it can be widely used. SELECT enables the quantitative detection of m6A by qPCR. Recently, glyoxal and nitrite-mediated deamination of unmethylated adenosine (GLORI) realized the high efficiency and unbiased detection of single base m6A site and the absolute quantification of the modification level of m6A [179]. It is conceptually similar to bisulfite-sequencing-based quantification of DNA 5-methylcytosine, in which inosine is formed by efficient deamination of unmethylated adenosine using a catalytic system of glyoxal and nitrite. Inosine is paired with cytidine in the reverse transcription process and then read as guanosine (G) in the sequencing process, resulting in an A-to-G transformation. m6A remained the same and still read as A after sequencing. GLORI realized the absolute quantification of single base m6A by detecting the proportion of A in the sequence of the read segment. SELECT [177], MeRIP-qPCR [167], and MazF-qPCR [170] are often used to detect and quantify the m6A level of m6A-containing fragments or genes.

In addition, Mass spectrometry or colorimetry detects the global m6A level of RNAs, whereas high-performance liquid chromatography can identify specific m6A residues [180]. Additionally, dot blot technology is often used to observe m6A changes whereas it cannot be utilized to quantify m6A and identify the exact m6A modification sites [71]. However, researchers can use other methods, such as RNA photo-cross and immunoprecipitation (PAR-CLIP) [181] and electrochemical immunosensor [182] to detect m6A instead of the precise m6A modification sites.

## DISCUSSION AND CONCLUSION

In this review, we summarized the physiological functions of m6A regulators, the m6A-related databases, existing predictors for m6A sites, methods for detecting m6A, and the interplays of m6A and mRNA as well as ncRNAs in cancers. Generally, m6A writers install methyl groups on the 6^th^ N of RNA adenosine whereas erasers remove the methylation. The readers recognize m6A sites of RNAs to regulate the translocation, splicing, degradation, stabilization, translation of RNAs, and other diverse biological functions. Interestingly, m6A may play oncogenic or suppressive roles in cancer initiation, progression, or resistance to therapy. How m6A affects cancer progression by regulating target genes depends on three factors: (1) whether the target gene acts as a tumor promoter or a tumor suppressor; (2) abnormal levels of m6A in cancer (depending on changes in writer or erase expression or activity); (3) regulation of target mRNA after methylation (as determined by reader). For instance, m6A on oncogenes can maintain their stabilities, MALAT1 in breast cancer or promote their degradation, XIST in CRC, as shown in Table 5. Tissue types play a more significant role in cancer genetics than previously realized, which should deserve consideration when designing treatments designed to inhibit cell proliferation. Well-designed bioinformatics analysis and mathematic modeling might be the key to elucidating the functional duality of m6A and maintaining physiological balance in different cellular contexts. The discrepancy among different research teams suggests that subsequent study requires shifting to a larger, multicenter paradigm to lay the foundation for the precision treatment of human tumors.

As emphasized above, m6A occurs on mRNA and ncRNAs and impacts the function of ncRNAs in cancers. For miRNAs, m6A affects the biogenesis and binding capacity with DGCR8 to participate in cancer progression. Concerning lncRNAs, m6A can function on lncRNAs to regulate their translocation, stabilization, and degradation, thereby leading to aberrant expression in cancers. Moreover, m6A acts on circRNA just like lncRNAs, except that m6A can also regulate the translation of circRNAs with coding ability. Conversely, lncRNAs and circRNAs can affect m6A levels directly by interacting with m6A regulators and indirectly by affecting m6A regulators through miRNAs, indicating that miRNAs can directly regulate the m6A by functioning on m6A regulators in cancers. This all suggests that m6A methylation on mRNAs or ncRNAs can be a potential target for cancer diagnosis, prognosis, and therapy. However, the investigation of the role of m6A in ncRNA remains lacking and needs further studies.

m6A regulators play controversially dual roles as oncogene or tumor suppressor genes across diverse cancers, which needs further exploration. And although accumulating evidence supports that m6A, mRNA, or ncRNA may be potential targets for cancer therapy, very few of them accomplish the clinical research stages, indicating a long way to go for clinical verification. In addition, there may be other RNA type-specific writers, readers, and erasers that have yet to be discovered, which will be crucial for gaining a better understanding of the function of m6A modification.

The rapid development of m6A detecting methods, m6A predicting methods trained by existing m6A modified sequences, and m6A-related databases significantly advance m6A investigation. Generally, Antibody-based m6A sequencing methods can preenrich for m6A signal and facilitate detection of low abundance RNA transcripts, but m6A-specific antibodies often have inherent bias and can also recognize other secondary modifications. On the contrary, chemical marker-based m6A sequencing technologies, such as m6A-SEAL and m6A-lable-seq can circumvent this problem. Endonuclease-based sequencing technologies such as MAZTER-seq and m6A-REF-seq utilize an endonuclease to detect m6A modification. However, due to the sequence bias of MazF, methods based on this enzyme can only detect part of the m6A site. Therefore, it is necessary to use these methods comprehensively in order to better detect m6A.

However, the explosion of multiple technologies has also increased confusion for researchers who lack explicit evaluations. Moreover, while mapping m6A was a critical advance, it required information on stoichiometry. New methods such as Mazter-Seq can accurately determine the stoichiometry of m6A at specific sites of particular mRNAs to determine if the transcriptome is truly dynamic and variable in different cellular and tumor progression contexts. Future studies should reveal to what extent m6A can dynamically regulate the epigenetic transcriptome of the tumor microenvironment and tumor-related immune environment to affect gene expression.
